# Hypoxia-mediated alterations and their role in the HER-2/neuregulated CREB status and localization

**DOI:** 10.18632/oncotarget.10474

**Published:** 2016-07-07

**Authors:** André Steven, Sandra Leisz, Katharina Sychra, Bernhard Hiebl, Claudia Wickenhauser, Dimitrios Mougiakakos, Rolf Kiessling, Carsten Denkert, Barbara Seliger

**Affiliations:** ^1^ Institute of Medical Immunology, Martin Luther University Halle-Wittenberg, Halle, Germany; ^2^ Centre for Basic Medical Research, Martin Luther University Halle-Wittenberg, Halle, Germany; ^3^ Institute of Pathology, Martin Luther University Halle-Wittenberg, Halle, Germany; ^4^ Department of Internal Medicine 5, Hematology and Oncology, University of Erlangen-Nuremberg, Erlangen, Germany; ^5^ Karolinska Institutet, CCK, Stockholm, Sweden; ^6^ Charité, Institute of Pathology, Berlin, Germany

**Keywords:** CREB, HER-2/neu, hypoxia, angiogenesis, mitochondria

## Abstract

The cAMP-responsive element-binding protein (CREB) is involved in the tumorigenicity of HER-2/neu-overexpressing murine and human tumor cells, but a link between the HER-2/neu-mediated CREB activation, its posttranslational modification and localization and changes in the cellular metabolism, due to an altered (tumor) microenvironment remains to be established. The present study demonstrated that shRNA-mediated silencing of CREB in HER-2/neu-transformed cells resulted in decreased tumor formation, which was associated with reduced angiogenesis, but increased necrotic and hypoxic areas in the tumor. Hypoxia induced pCREB^Ser133^, but not pCREB^Ser121^ expression in HER-2/neu-transformed cells. This was accompanied by upregulation of the hypoxia-inducible genes GLUT1 and VEGF, increased cell migration and matrix metalloproteinase-mediated invasion. Treatment of HER-2/neu^+^ cells with signal transduction inhibitors targeting in particular HER-2/neu was able to revert hypoxia-controlled CREB activation. In addition to changes in the phosphorylation, hypoxic response of HER-2/neu^+^ cells caused a transient ubiquitination and SUMOylation as well as a co-localization of nuclear CREB to the mitochondrial matrix. A mitochondrial localization of CREB was also demonstrated in hypoxic areas of HER-2/neu^+^ mammary carcinoma lesions. This was accompanied by an altered gene expression pattern, activity and metabolism of mitochondria leading to an increased respiratory rate, oxidative phosphorylation and mitochondrial membrane potential and consequently to an enhanced apoptosis and reduced cell viability. These data suggest that the HER-2/neu-mediated CREB activation caused by a hypoxic tumor microenvironment contributes to the neoplastic phenotype of HER-2/neu^+^ cells at various levels.

## INTRODUCTION

The cAMP-responsive element-binding protein (CREB) belongs to the leucine zipper family of transcription factors. Different mediators, such as growth factors, neurotransmitters, glucose, inflammatory lipids, stress signals as well as factors known to modulate the intracellular cAMP or Ca^2+^ levels can activate CREB through phosphorylation of serine 133 (Ser^133^) by protein kinase A (PKA), protein kinase B (PKB/AKT), mitogen-activated protein kinases (MAPK), calcium-activated calmodulin kinases (CaMKs) and the 90 kD ribosomal S6 kinase. Stress situations like irradiation and UV caused phosphorylation of CREB at serine 121 (Ser^121^) leading to its activation [1–3], while phosphorylation of CREB at Ser^142^ could negatively interfere with CREB activity [2]. The phosphorylated CREB (pCREB) can bind to its transcriptional co-activator (CBP/p300) via the kinase-induced domain (KID) in the cAMP- (CRE) and the KID-interacting domain KIX of CBP [4]. The CREB/CBP complex recruits the transcription machinery to the gene promoter thereby initiating CRE-regulated gene transcription. Upon activation CREB regulates the expression of many genes involved in different physiologic and pathophysiologic processes by binding to the CRE sequence within their promoters [5–9].

In addition, pCREB has been shown to be present and active in mitochondria (mitoCREB): This not only leads to an altered expression pattern of mitochondrial genes [10–13], but also to changes in the mitochondrial function, which could affect cell growth and survival. Although phosphorylation of CREB is the most studied modification, other post-transcriptional modifications (PTMs), like ubiquitination, acetylation and SUMOylation, could also exist. However, the frequency and role of these PTMs for CREB expression and function are incompletely understood [14–17]. Next to the phosphorylation-dependent activation of CREB, the stability of CREB is influenced by hypoxia. In the acute phase of hypoxia CREB can be ubiquitinated and degraded via the ubiquitin/proteasome pathway [18, 19], while SUMOylation of CREB is increased during prolonged hypoxia. The hypoxia-mediated stabilization and activation of CREB results in the induction of various genes [20] leading to an adaptation to hypoxia coupled with a translocation of CREB from the nucleus to the mitochondria [21]. This process caused changes of the mitochondrial function and biogenesis thereby modulating cellular processes including cell survival [13, 22].

A role for CREB in the development of tumors has been suggested since it is often overexpressed and more active in many solid and hematopoietic tumors [23–26]. This could be influenced by the modulation of signalling cascades upstream of CREB as well as the induction of the CRE-dependent gene expression downstream of CREB, e.g. matrix metalloproteinases (MMPs), adhesion molecules and different survival factors [20, 27] leading to an increased tumor growth, prevention of cell death, enhanced metastasis formation and angiogenesis [28]. Furthermore, overexpression of CREB in tumors often correlates with disease progression, poor patient survival and chemotherapy resistance [21, 24–26, 29–31]. On the other hand oxygenation in breast cancer is markedly reduced compared to normal tissue [32]. In mammary carcinoma the CREB pathway is often upregulated [33, 34]. An important role of CREB in breast cancer has been further demonstrated by global profiling of signalling networks in breast cancer stem cells [34]. Recently, a correlation between CREB expression/activation, altered *in vitro* and *in vivo* tumor growth properties and HER-2/neu has been described in both *in vitro* models of HER-2/neu transformation and in HER-2/neu overexpressing human mammary carcinoma [35]. Despite the tumor microenvironment including hypoxia is of critical importance for breast cancer [36], it has not yet been determined whether the HER-2/neu-mediated expression, activation, localization and modification of CREB and upstream signal pathways are altered under hypoxic conditions. Therefore this study analysed the changes of the cellular localization and posttranslational modifications in different HER-2/neu model systems under normoxia and hypoxia in the presence of signal transduction inhibitors.

## RESULTS

### Link between decreased tumorigenicity of CREB-deficient cells and reduced angiogenesis

**Figure 1:**
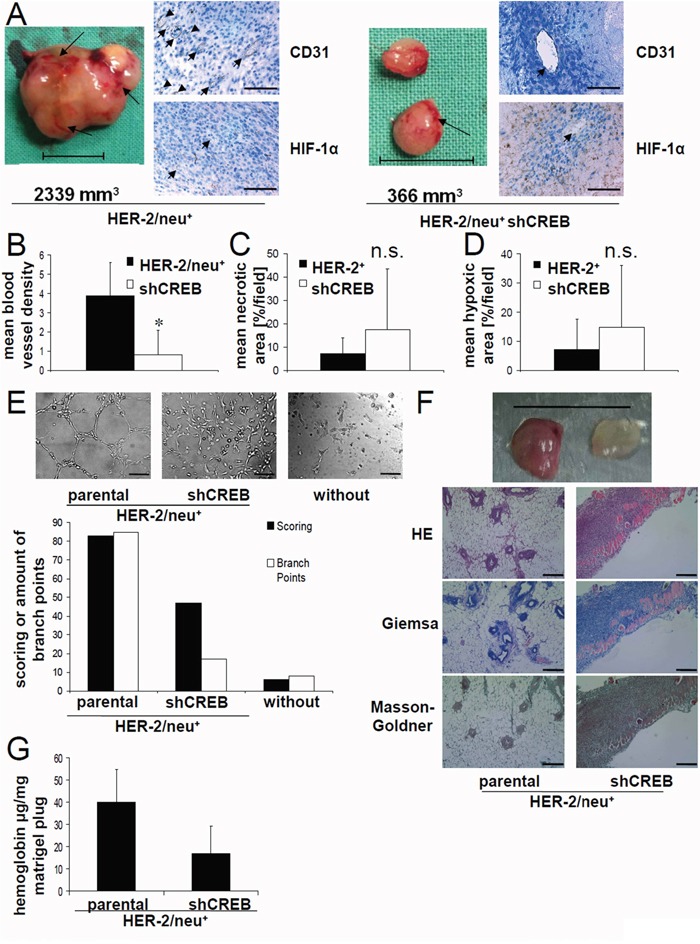
Link of decreased tumorgenicity of CREB-deficient HER-2/neu^+^ cells and reduced angiogenesis, but enhanced hypoxic areas **A.** DBA-1 mice were injected with parental or CREB-deficient HER-2/neu^+^ cells as described in Materials and Methods and tumors were removed after 42 days. Representative photos of parental and CREB-deficient HER-2/neu^+^ tumors are shown. The arrows indicate the blood vessels on the tumor surface. The tumor volume is given. The bar represents 1 cm (left). 5 μm slices of paraffin-embedded tumors were stained with the indicated primary antibody followed by an anti-rabbit secondary antibody. The detection was performed with the peroxidase substrate DAB. Slides were counterstained with methylene blue. The arrow heads indicate the blood vessels. The bar represents 100 μm; Magnification: 40x (right). **B.** The blood vessel density of the tumors was analysed by counting vessel structures in the anti-CD31 mAb-stained samples (see 1A). Bars represent mean values from four samples/group with four counted fields/sample. **C.** The necrotic area was analysed in the HE-stained samples. Bars represent mean values from four samples/group with four counted fields/sample. **D.** The hypoxic area was analysed in the anti-HIF-1α-stained samples. Bars represent mean values from four samples/group with four counted fields/sample. **E.** 1×10^4^ HUVEC/well were seeded in a 96 well plate on polymerized growth factor reduced matrigel. 100 μl/well fresh medium or cell conditioned medium was added and the cells were incubated for 16 h by 37°C. The morphology of the HUVEC under these distinct culture conditions was compared (left) and the mesh-like structures were quantified (right) as described by Zhang [51]. The bar represents 80 μm; Magnification: 10x. **F.** 1×10^5^ parental and CREB-deficient HER-2/neu^+^ cells resuspended in matrigel were injected into the flank of female DBA-1 mice (n = 8). 7 days after injection the mice were killed and the removed matrigel plugs were photographed. The bar represents 1 cm (up). 5 μm slices of the matrigel plugs from parental and CREB-deficient HER-2/neu^+^ cells were stained as indicated. The bar represents 100 μm; Magnification: 10x (down). **G.** Matrigel plugs from parental and CREB-deficient HER-2/neu^+^ cells were homogenized and their hemoglobin content was analysed as described as in Material and Methods. The bars represent the hemoglobin concentration of each plug from mice injected with the indicated cell line normalized to the weight of the plug. Data demonstrate the results of one out of two independent experiments (with five Matrigel plugs in each experiments) regarding the hemoglobin content/plug from parental and CREB-deficient HER-2/neu^+^ cells.

Recently, a link between HER-2/neu overexpression and CREB activation has been described in parental HER-2/neu^+^ cells upon silencing of CREB by shRNA without affecting HER-2/neu surface expression [35]. This was accompanied by a decreased tumor growth of CREB-deficient compared to parental HER-2/neu^+^ cells ([35], Supplementary Figure 1A). In order to determine whether the diminished *in vivo* growth capacity of CREB-deficient HER-2/neu^+^ cells was associated with a reduced angiogenesis, lesions of parental and shCREB HER-2/neu^+^ murine tumors were stained with anti-CD31 and anti-HIF-1α antibodies, respectively. As shown in Figure 1A, an altered staining pattern for CD31 and HIF-1α was demonstrated in parental versus CREB-deficient HER-2/neu^+^ cells with a more than 50% reduced density of blood vessels (Figure 1B), an approximately 2-fold increase of necrotic (Figure 1C) as well as hypoxic areas (Figure 1D) in CREB-deficient tumors when compared to parental HER-2/neu^+^ tumors. It is noteworthy that the increased HIF-1α expression has been correlated with a worse prognosis of breast cancer patients, while CREB increased the risk of metastases in HER-2/neu^+^ breast cancer (Supplementary Figure 2 and Supplementary Table 1).

The CREB-mediated regulation of angiogenesis was confirmed *in vitro* by incubating human umbilical vein endothelial cells (HUVEC) with conditioned medium obtained from both parental and CREB-deficient HER-2/neu^+^ cells. In the presence of supernatants from parental HER-2/neu^+^ cells morphologic changes of HUVEC were more pronounced with increased numbers of branching points when compared to that from CREB-deficient HER-2/neu^+^ cells (Figure 1E). To confirm the angiogenic potential of CREB, both parental and CREB-deficient HER-2/neu^+^ cells were injected with matrigel into the flanks of mice followed by the analysis of tumor growth, hemoglobin content and blood vesel density in these tumors. The matrigel plugs of CREB-deficient HER-2/neu^+^ cells had a smaller size, a fainter red color (Figure 1F), reduced hemoglobin content and decreased number of blood vessels (Figure 1G). This was accompanied by a downregulation of the mRNA expression levels of VEGF-A and ICAM-1 in CREB deficient cells when compared with parental HER-2/neu^+^ cells (Table 1).

**Table 1:** Influence of CREB knock down on the expression of surface and angiogenesis markers

	NIH3T3	NIH3T3 shCREB	HER-2/neu^+^	HER-2/neu^+^ shCREB
Angiopoietin 1	1	0.56 +/− 0.23	4.06 +/− 3.01	4.63 +/− 0.41
Angiopoietin 2	1	1.7 +/− 0.39 *	0.65 +/− 0.7	1.45 +/− 0.44
ICAM-1	1	0.82 +/− 0.5	0.12 +/− 0.03	2.07 +/− 1.2 *
PECAM1	1	1.08 +/− 0.54	0.28 +/− 0.34	0.29 +/− 0.23
VCAM1	1	0.56 +/− 0.49	3.62 +/− 5.63	0.18 +/− 0.02
VEGF-A	1	0.87 +/− 0.53	2.76 +/− 0.31	0.79 +/− 0.49 *

Cellular RNA isolated from parental and CREB-deficient (shCREB) HER-2/neu^−^ and HER-2/neu^+^ cells was subjected to qPCR analysis as described in Materials and Methods. Using gene-specific primers the expression of two angiogenesis markers (VEGF, angiopoetin 1) as well as two adhesion molecules linked with angiogenesis (ICAM-1, VCAM1) was determined. The results represent the mean and SEM from three independent replicates. The expression was normalized to β-actin and the data are expressed as relative mRNA expression by setting the expression levels of NIH3T3 cells to 1.

### Determination of the role of hypoxia on the expression and activation of CREB and its dependence on the HER-2/neu-mediated signaling

**Figure 2:**
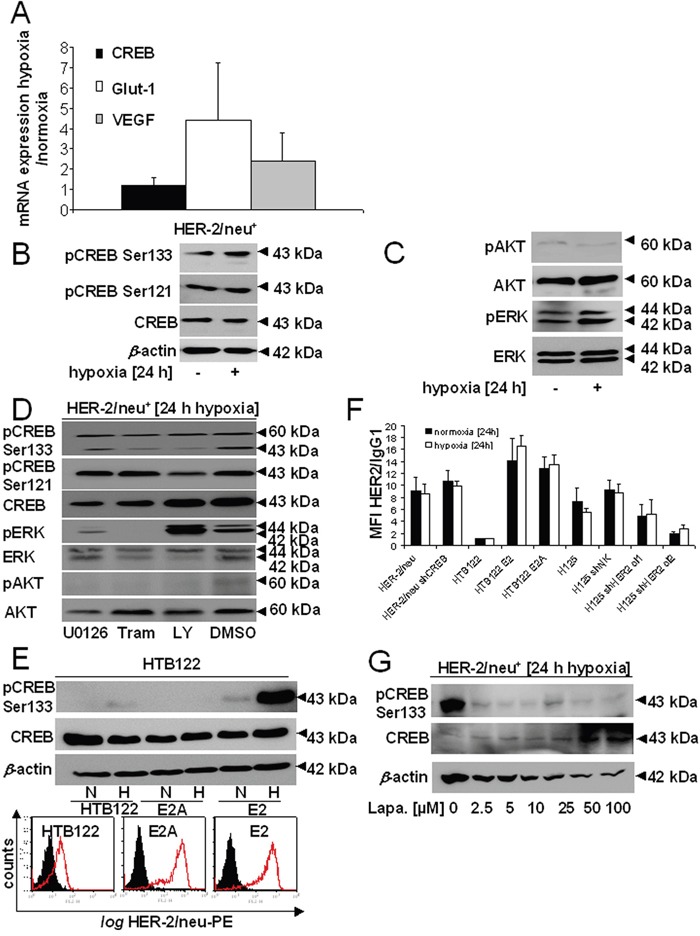
Hypoxia-induced CREB phosphorylation by induction of the MAPK/ERK signal transduction pathway **A.** The transcription of CREB and of hypoxic markers (GLUT1, VEGF) was analysed by qPCR. The bar charts represent the mean values and SEM of three independent experiments. **B.** CREB expression and phosphorylation was compared in HER-2/neu^+^ NIH3T3 cells under normoxia and hypoxia by Western blot analysis as described in Materials and Methods using an anti-CREB and anti-CREB phosphorylation-specific antibodies. One of three representative Western blots is shown. **C.** The activity/phosphorylation of the AKT and ERK pathway was determined under normoxic and hypoxic conditions by Western blot analysis using total and phosphorylation-specific antibodies, respectively. The data represent one of three biological replicates. **D.** Cells were treated under hypoxia with 5 μM LY294002, 100 nM trametinib or 5 μM U0126 for 24 h. The phosphorylation and total expression of CREB, AKT and ERK was analysed by Western Blot. **E.** Hypoxia-mediated induction of CREB phosphorylation and its dependence on the HER-2/neu status was determined in HTB122 cells and their HER-2/neu-transformed transfectants (E2A: dominant negative mutation in the HER-2/neu kinase domain; E2: wild-type HER-2/neu) either incubated under normoxia or hypoxia for 24 h, respectively, before Western blot analysis was performed as described in Materials and Methods. The results show one of two independent experiments (up). HTB122, E2 and E2A cells were incubated under normoxic conditions for 24 h before the HER-2/neu cell surface expression was determined using flow cytometry. The data are represented as histograms from one out of two representative experiments. The black area represents the IgG control, while the red defined area is the HER-2/neu-PE staining (down). **F.** Cells were incubated for 24 h under normoxia or hypoxia and the presentation of HER-2/neu on the cell surface was determined by flow cytometry as described in Materials and Methods using a PE-labelled anti-HER-/neu mAb. The bars represent the MFI of HER-2/neu compared to an IgG control from two independent experiments. **G.** The effect of inhibition of the HER-2/neu activity by treatment of parental HER-2/neu^+^ cells with increasing concentrations of lapatinib on CREB phosphorylation was analysed by Western blot. Cells were treated with lapatinib for 24 h under hypoxic conditions.

Since an oxygen-limited microenvironment (hypoxia) has recently been shown to activate CREB [18, 20], the expression level and phosphorylation status of CREB was compared in HER-2/neu^−^ NIH3T3 cells and parental HER-2/neu^+^ cells cultured under normoxic and hypoxic conditions. The induction of hypoxia in these cells was confirmed by an upregulated transcription of VEGF-A and the glucose transporter (GLUT)1 (Figure 2A).

The expression and activation of CREB in the model systems was found to be different both under normoxic and hypoxic culture conditions. A strong upregulation of unphosphorylated CREB and pCREB^Ser133^ was found in hypoxic HER-2/neu^+^, while expression of pCREB^Ser121^ was unchanged after 24 h hypoxia in these cells (Figure 2B). The increased phosphorylation status of CREB at Ser^133^ was accompanied by an upregulated expression of pERK, but not of ERK, AKT or pAKT in the HER-2/neu^+^ cell line (Figure 2C), while the cAMP levels were not changed under hypoxic conditions (data not shown) resulting in an unaltered PKA activity. Treatment with the MEK inhibitor trametinib decreased pCREB^Ser133^ but not pCREB^Ser121^ while 5 μM U0126 had no effect (Figure 2D). Blocking PI3K/AKT by LY294002 reduced the phosphorylation of both serine residues. There might be a minor effect of U0126 on the expression of AKT, which does not have an impact on the general results. Analogous to murine parental HER-2/neu^+^ cells, hypoxia increased pCREB^Ser133^ expression in the breast cancer cell line HTB122 transfected with functional HER-2/neu (E2), but not in HTB122 cells transformed with a signaling-deficient HER-2/neu construct (E2A, Figure 2E). Vice versa the shHER-2/neu-transfected lung carcinoma cell line H125 exhibited a downregulation of pCREB^Ser133^ expression under hypoxic conditions (Supplementary Figure 1B). These data suggested a control of CREB activity by both oncogenic transformation and hypoxia. It is noteworthy that hypoxia did not influence the HER-2/neu surface expression in murine and human HER-2/neu-transformed cells (Figure 2F).

In order to determine whether inhibitors of transduction pathways affect the hypoxia-mediated expression of CREB, hypoxic parental HER-2/neu^+^ cells were treated with various inhibitors followed by the analysis of the phosphorylation status of CREB. As shown in Figure 2G the tyrosine kinase inhibitor (TKI) lapatinib targeting HER-1 and HER-2/neu had the most significant impact and already 2.5 μM lapatinib caused an 80% downregulation of the expression of pCREB^Ser133^, which could not be further decreased by higher concentrations. In contrast, lapatinib treatment consecutively increased the total CREB expression in a dose-dependent manner.

### Hypoxia-induced post-translational modifications of CREB

**Figure 3:**
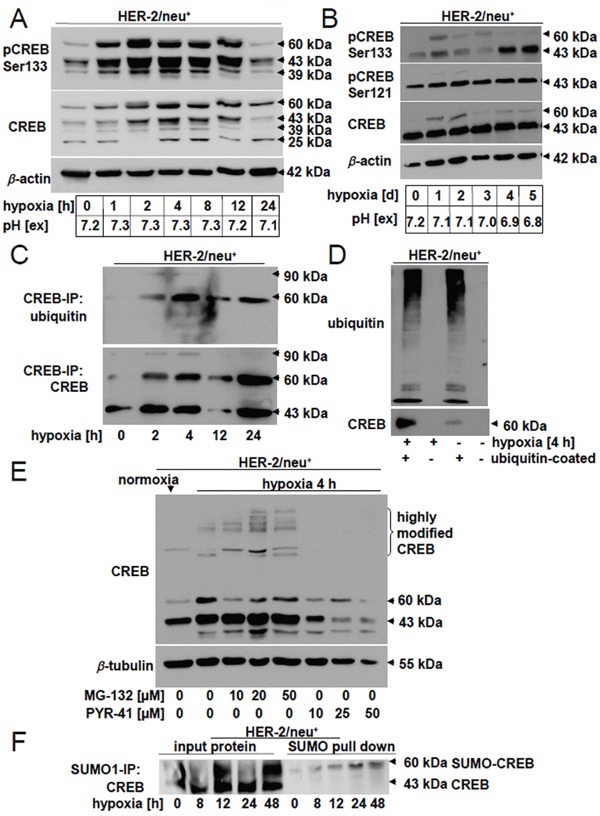
Hypoxia-mediated post-transcriptional modification and altered distribution of CREB **A.** Comparative analysis of different pCREB and CREB modifications under normoxia and early phase hypoxia (up to 12 h). Western blot analyses were performed as described using CREB and pCREB^Ser133^-specific mAb. The size of the modified CREB protein is given. The blots represent one of three biological replicates. Extracellular pH (pH [ex]) of the cell free media was directly measured with a pH electrode after harvesting the cells. **B.** CREB phosphorylation at serine residue 133 and 121 as well as CREB protein expression was analysed under late phase hypoxia (up to 5 d). The photos represent one of two independent biological replicates. The pH of the culture media was measured with a pH electrode directy after harvesting the cells. **C.**, **D.** Ubiquitination was analysed by CREB immune precipitation (C) and ubiquitin pull down (D) as described in Materials and Methods by loading the complete supernatant on 10% (C) or 12% (D) gels. The proteins were identified by using anti-CREB- or anti-ubiquitin-specific mAb. Results represent data of three (C) or two (D) biological replicates. **E.** Influence of the proteasome inhibitor MG-132 and the ubiquitin inhibitor PYR-41 on CREB modifications. Parental HER-2/neu^+^ cells were either left untreated or treated with different concentrations of MG-132 (10, 25, 50 μM) or PYR-41 (10, 20, 50 μM) for 4 h under hypoxia. Following Western blot analysis using anti-CREB-specific antibodies as described above, the appearance of highly modified CREB molecules was determined. The blot represents one of two biological experiments. **F.** Cells were cultivated under hypoxia for the indicated time and SUMO-1 modified proteins in the cell lysate were precipitated by immunoprecipitation. The proteins were loaded onto a 10% SDS Gel and CREB was detected with specific antibodies.

In order to dissect the effect of early and late phase hypoxia on the CREB status and its PTMs, time kinetic analyses of parental HER-2/neu^+^ cells were performed. A transient, but time-dependent upregulation of CREB and pCREB^Ser133^, but not of pCREB^Ser121^ was detected during hypoxia: Already at one hour after hypoxia onset a significant increase in both CREB and pCREB^Ser133^ protein expression was found (Figure 3A). This was accompanied by a transient induction of pCREB^Ser133^ modifications in these cells during the early phase of hypoxia with the highest levels at 2 h and their subsequent decline upon prolonged hypoxia (late phase) (Figure 3B). A comparable phosphorylation pattern, but modified CREB forms as demonstrated by two bands at ~90 and ~120 kDa were observed in the HER-2/neu^+^ breast cancer cell line SKBR3 under hypoxic treatment (Supplementary Figure 1C), while the 60 kDa band was neither visible with CREB nor with pCREB antibodies. In the early phase of hypoxia (up to 48 h) no changes in the pH were measured, but a sligthly more acid pH was observed after 5 d.

Immunoprecipitation with an anti-CREB-specific antibody demonstrated a hypoxia-induced ubiquitination of CREB in parental HER-2/neu^+^ cells (Figure 3C), which was also confirmed by an ubiquitin pull down assay (Figure 3D). The role of the ubiquitin-dependent proteasomal degradation for CREB expression was further analysed in hypoxic parental HER-2/neu^+^ cells either left untreated or treated with increasing concentrations of the ubiquitin inhibitor PYR-41 (10, 25, 50 μM) and of the proteasome inhibitor MG-132 (10, 20, 50 μM), respectively. Treatment of hypoxic parental HER-2/neu^+^ cells with 10 μM PYR-41 abrogated the expression of highly modified CREB, whereas total CREB protein expression was reduced (Figure 3E). In contrast, highly modified CREB expression levels were enhanced in hypoxic cells with increasing concentrations of MG-132. Furthermore, immunoprecipitation of SUMO-1 followed by the detection with CREB-specific antibodies revealed a SUMOylation of CREB after 8 h hypoxia, which further increased during 48 h hypoxia (Figure 3F).

### Hypoxia-induced changes in the localization of CREB in HER-2/neu^+^ cells

**Figure 4:**
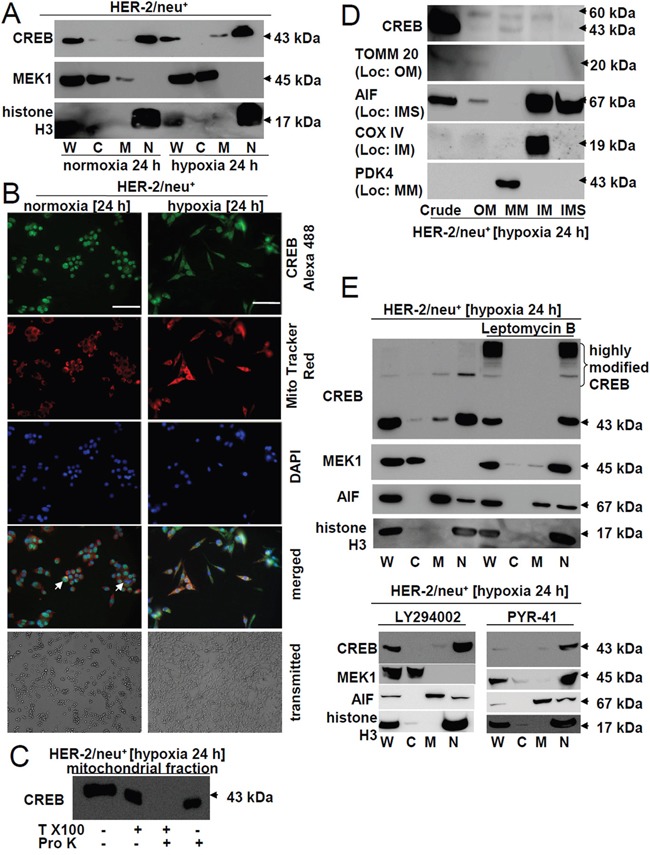
Increased mitochondrial localization of CREB under hypoxia **A.** The distribution of CREB in different cell fractions was analysed by using a commercial kit for separations followed by Western blot analysis of the distinct fractions. Anti-MEK1 (cytosol) and anti-histone H3 (nucleus) mAb served as markers for the subcellular compartments. “W” marks the whole cell lysate, “C” the cytosolic fraction, “M” the membrane/mitochondrial fraction and “N” the nuclear fraction. The picture represents one of three independent experiments. **B.** Cells were incubated for 24 h under hypoxia or normoxia and 30 min before terminating the cells were stained with 500 nM MitoTracker. After removing the media and fixation with 4% PFA for 20 min the cells were permeabilized with 0.5% Triton X100 in HBBS (HBSS-T) for 30 min. Following incubation with CREB antibody overnight at 4°C the cells were washed three times with HBSS, incubated with the secondary antibody (rabbit-Alexa 488) for 1 h, washed again three times with HBSS and then the nuclei were stained with DAPI. Single and merged colors recorded at a Pathway 855 (BD) are shown. Note the complete loss of nuclear CREB in dividing cells (white arrow heads). The bar represents 100 μm; Magnification: 20x. Morphology of the cells cultivated under normoxia or hypoxia (24 h each) was recorded by microscopic pictures. The pictures were taken on living, non-stained cells (transmitted). **C.** 1 mg isolated intact mitochondria were incubated in 1% Triton X100 and/or 10 U proteinase K in proteinase K buffer and were incubated at 37°C for 30 min and 60°C for 10 min. The reaction was stopped by boiling the probes in Laemmli buffer for 5 min and the stability of CREB was analysed by Western blot using an anti-CREB-specific mAb as described in Materials and Methods. **D.** Mitochondria were sub fractionated into outer membrane (OM), inter membrane space (IMS), inner membrane (IM) and mitochondrial matrix (MM). The purity of the fractions was analysed with marker proteins: TOM20 for OM, AIF for IMS, COXIV for IM, PDK4 for MM. **E.** Cells were cultured in the presence or absence of 50 ng/ml Leptomycin B, 10 μM LY294002 and 10 μM PYR-41 for 24 h under hypoxic conditions. Then cellular proteins were fractionated as described in the Material and Methods section. Additionally to the marker proteins used in (A) AIF served as a marker for the mitochondrial fraction. “W” represents the whole cell lysate, “C” the cytosolic fraction, “M” the membrane/mitochondrial fraction and “N” the nuclear fraction. The picture shows one of two experiments. An accumulation of highly modified CREB and the nuclear translocation of MEK1 and partially of AIF was found under leptomycin B treatment.

Since stress conditions can cause a translocation of nuclear CREB into mitochondria (mito-CREB) thereby altering the expression of mitochondrial genes including oxidative phosphorylation complexes [7, 37, 38], the subcellular localization of CREB was determined in parental HER-2/neu^+^ cells under normoxic and hypoxic conditions. As shown in Figure 4A subcellular fractionation revealed a partly translocation of CREB from the nucleus to the mitochondria in hypoxic parental HER-2/neu^+^ cells. Western blot analysis using antibodies directed against the cytosolic (MEK1) or nuclear (histone H3) compartment served as controls (Figure 4A). These *in vitro* data were confirmed by immunohistochemical analysis of mammary carcinoma lesions demonstrating an increased extracellular localization of pCREB in hypoxic breast cancer areas (Supplementary Figure 3).

The specificity of mitochondrial CREB in hypoxic parental HER-2/neu^+^ cells was confirmed using the mitochondria-specific dye MitoTracker. A co-localization of CREB into mitochondria of hypoxic, but not of normoxic parental HER-2/neu^+^ cells was detected with this dye (Figure 4B, ICC pictures), which was accompanied by morphologic changes of hypoxic parental HER-2/neu^+^ cells towards a more spindle-shaped phenotype (Figure 4B, transmitted light). Under hypoxic conditions nearly 30% of CREB was extra-nuclear localized when compared to ~ 5% under normoxic conditions. The translocation of CREB into mitochondria (mito-CREB) was confirmed by its resistance to digestion with trypsin unless the mitochondria were dissolved by Triton X100 (Figure 4C). Upon sub-fractionating and validation of the purity of the different mitochondrial fractions using specific markers CREB expression was found in the mitochondrial matrix, while the modified form of CREB could be detected in the matrix as well as in the inner and outer mitochondrial membrane (Figure 4D).

The nuclear export of CREB was further shown by treatment of cells with the nuclear translocation blocker leptomycin B. A combination of hypoxia and treatment with 50 ng/ml leptomycin B for 24 h totally abrogated the expression of mito-CREB in parental HER-2/neu^+^ cells and significantly enriched high molecular weight forms of CREB in the nucleus (Figure 4E). In the presence of leptomycin B the expression of MEK1 shifted in parental HER-2/neu^+^ cells from the cytosol to the nucleus. A similar CREB expression pattern was detected in the presence of the PI3K inhibitor LY294002 and the E3 ubiquitin ligase inhibitor PYR-41 (Figure 4E).

### Changes in the biogenesis of parental vs. CREB-deficient HER-2/neu^+^ cells

**Figure 5:**
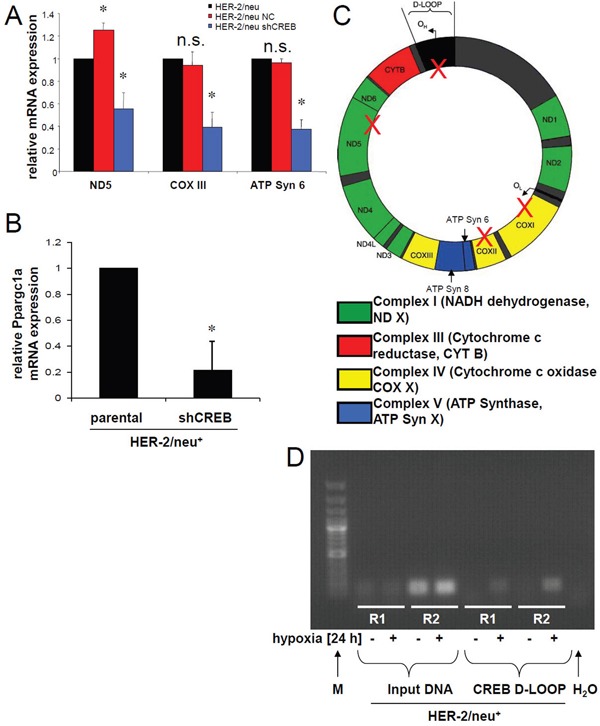
Regulation of the mitochondrial biogenesis via Ppar1a and CREB binding to mitochondrial elements **A.** mRNA expression of mitochondrial encoded genes was determined by real time PCR using gene specific primer. The bar charts represent the data from two independent experiments. **B.** Expression of Ppargc1a mRNA was quantified by qPCR. The bar charts represent the data from two independent experiments. **C.** Putative CRE motifs were identified in the mtDNA. The mtD-LOOP has 2 CRE elements and in the protein coding regions 3 other CRE elements were identified. These were marked with a red cross. **D.** CREB binding DNA was immunoprecipitated, purified and used in a real time PCR. The products were further analysed by 2% agarose gel electrophoresis. IgG rabbit antibody was used as a control. The gel represents two of three independent experiments.

In order to determine CREB function in the mitochondria the expression of the mitochondrial genes was compared in parental and CREB-deficient HER-2/neu^+^ cells. As shown in Figure 5A, the mitochondrial genes ND5, COXIII and ATP synthase 6 were downregulated in CREB-deficient HER-2/neu^+^ cells, whereas the expression of the mitochondrial genes ND1 – 4, ND4L, COXI, COXII and ATP synthase 8 remained unaltered in parental and CREB-deficient HER-2/neu^+^ cells (data not shown). Changes in the expression pattern of selected mitochondrial genes were not due to alterations of the mitochondrial (mt) DNA content (data not shown). The decreased mitochondrial biogenesis, respiration or incomplete mitophagy was further confirmed by the shCREB-mediated reduction of Ppargc1a transcription (Figure 5B). Since mtDNA contains several putative CRE sites (Figure 5C) the binding of CREB to the D-LOOP of the mtDNA was analyzed, which was enhanced under hypoxia (Figure 5D).

### Hypoxia-mediated changes of the mitochondrial metabolism of parental vs. CREB-deficient HER-2/neu^+^ cells

**Figure 6:**
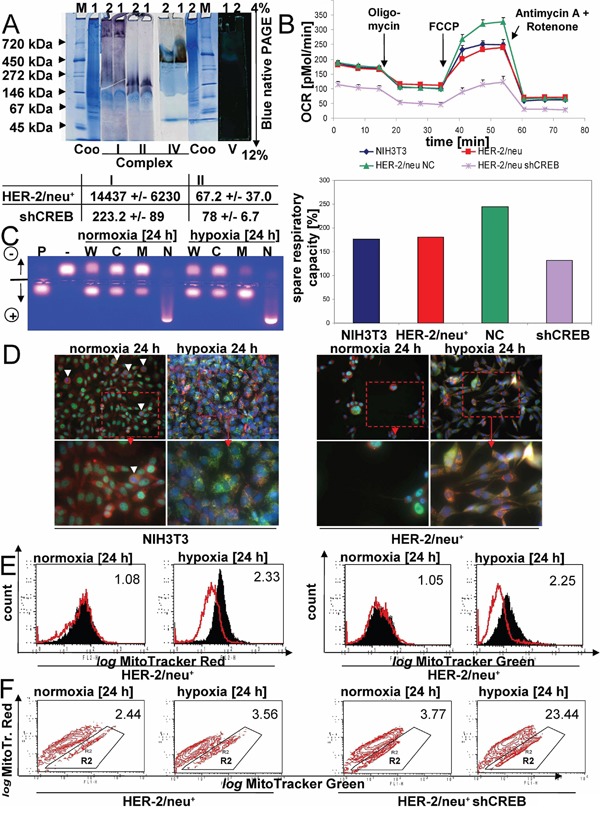
Regulation of mitochondrial functions by mito-CREB **A.** The activity of mitochondrial complexes was analysed by in gel activity. The numbers in A are the mitochondrial proteins from HER-2/neu^+^ cells (1) and HER-2/neu^+^ shCREB cells (2). M: representative molecular weight marker. Coo: Colloidal coomassie staining. The staining of complex V (ATP synthase) was documented in front of a dark background. The data beyond the gel staining represents the spectrometrically analysis of the complex I and II activities from three independent experiments. **B.** Basal oxygen consumption rate (OCR) (an indicator for mitochondrial respiration) was detected using the XF96e Extracellular Flux Analyzer (Seahorse Bioscience). Next, OCR responses towards the application of oligomycin (1 μM), FCCP (2.5 μM), and the combination of antimycin (3 μM), and rotenone (3 μM) (XF Cell Mito Stress Test Kit, Seahorse Bioscience) were evaluated. All experiments were performed in at least hexaplicates. Changes after FCCP application are indicative for the maximal respiratory capacity (up). The spare respiratory capacity was calculated from the results (down). **C.** The PKA activity of the whole cell lysate (W) and the intracellular fraction (C, M, N) was analysed as described in Materials and Methods. Samples (cultivated for 24 h under normoxia or hypoxia), positive (P) and negative (−) controls were loaded onto an agarose gel. The gel was photographed under UV irradiation. **D.** The localization of CREB (green), mitochondria (red) and the nucleus (blue) was compared in NIH3T3 and HER-2/neu^+^ cells under normoxia and hypoxia. White arrows mark dividing cells, which lacks nuclear CREB. Under hypoxic conditions the mitochondrial fission is visible. **E.** HER-2/neu^+^ cells and CREB-deficient derivatives were incubated under normoxia and hypoxia for 24 hours, before cells were stained with MitoTracker Green and Red, respectively. **F.** In the contour plot the cells in R2 represents mitochondrial dysfunctional cells (pos. for MitoTracker Green, weaker staining for MitoTracker Red). 10.000 cells were analysed by flow cytometry.

The mitochondrial fitness was further investigated by determining the activity of mitochondrial complexes in normoxic and hypoxic CREB-deficient/parental HER-2/neu^+^ cells. As shown in Figure 6A, NADH dehydrogenase activity (complex I) and complex IV was reduced in hypoxic CREB-deficient HER-2/neu^+^ cells, which is in line with the cell viability as well as the generation and maintenance of an electrochemical gradient. The increased mitochondrial metabolism/oxidative phosphorylation of parental HER-2/neu^+^ cells was further underlined by an increased basal respiratory rate (Figure 6B). In addition, the cells' maximal respiratory capacity was determined. Upon addition of the uncoupling agent FCCP a significantly stronger relative increase of the oxygen consumption rate (OCR) in parental HER-2/neu^+^ cells when compared to CREB-deficient HER-2/neu^+^ cells was detected. Furthermore, the increased dependency on maintaining the mitochondrial energetic flux was highlighted by the effects of parental HER-2/neu^+^ cells upon sequential treatment with oligomycin and FCCP, which blocked the mitochondrial function (Figure 6B). Furthermore, shCREB cells had an increased reliance on respiration for producing ATP through ATPase activity. Since PKA was more active in mitochondria upon hypoxia (Figure 6C), a PKA-dependent process was suggested, that can regulate the binding of mitochondrial CREB to the D-LOOP under hypoxia (Figure 5D).

When compared to normoxia a change in the mitochondrial morphology was found in the presence of hypoxia in both parental HER-2/neu^−^ and HER-2/neu^+^ cells. Mitochondria undergo mitochondrial fission caused by the accumulation of perinuclear mitochondria (Figure 6D, Supplementary Figure 4) and degradation of dysfunctional mitochondria by mitophagy. To further evaluate the mitochondrial function/dysfunction two types of mitochondria-specific dyes were used that distinguish respiring (MitoTracker Red) from total (MitoTracker Green) mitochondria. HER-2/neu^+^ cells displayed a stronger signal with MitoTracker Green under hypoxic but not under normoxic conditions when compared to their HER-2/neu^+^ shCREB counterparts (Figure 6E). Furthermore, parental HER-2/neu^+^ cells had an increased fraction of respiring (i.e. functional) mitochondria (Figure 6F) when compared to CREB-deficient HER-2/neu^+^ cells suggesting an enrichment of dysfunctional mitochondria in CREB-deficient cells under hypoxia, which is in line with our functional data for the mitochondrial biogenesis shown in Figure 5B.

### Functional consequences of hypoxia on CREB activity

**Figure 7:**
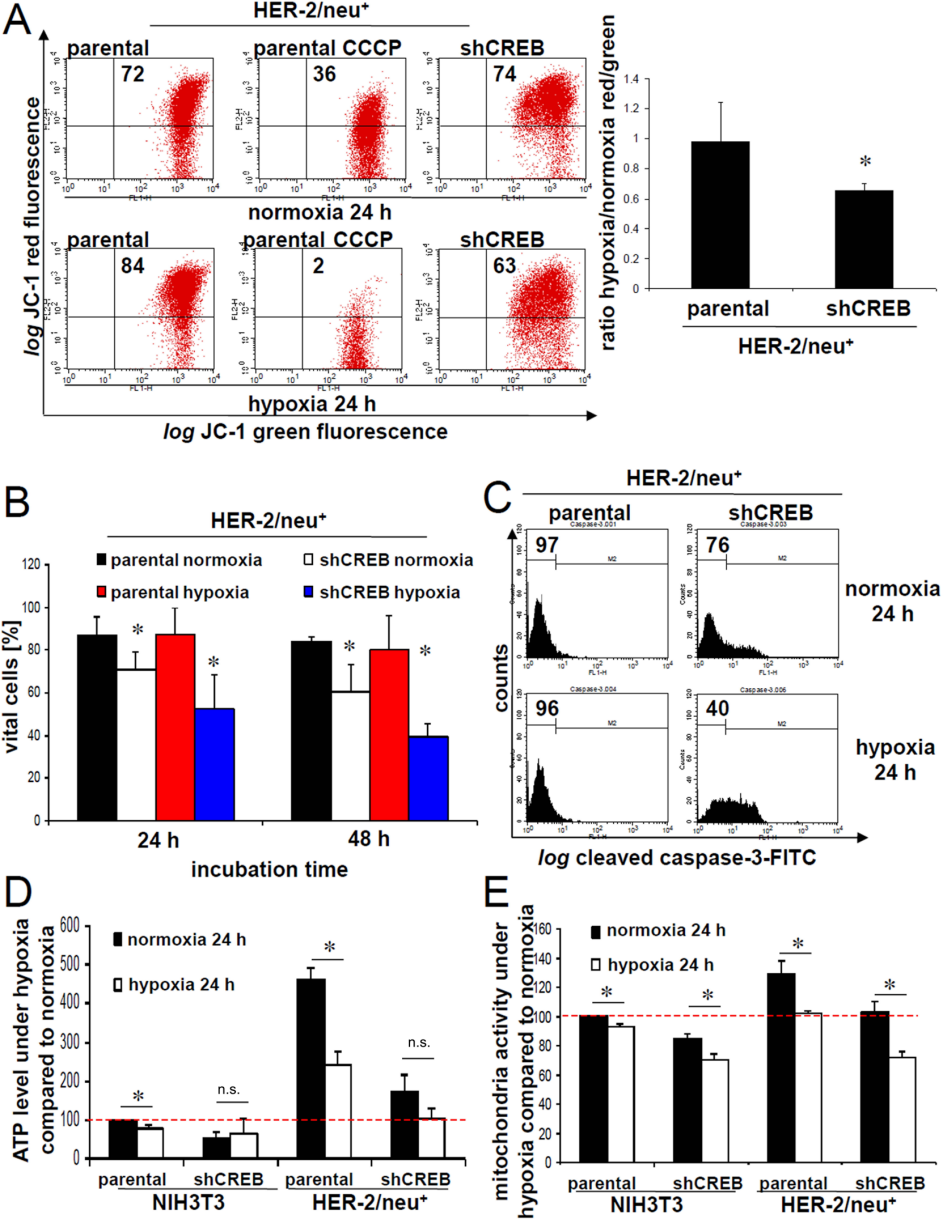
Decreased mitochondrial membrane potential and cell vitality upon CREB silencing **A.** The mitochondrial membrane potential was determined in normoxic or hypoxic cultivated cells by using JC-1 fluorescence analysis. The fluorescence of 5×10^3^ stained cells was analysed with a FACsCalibur (BD) and CCCP-treated cells (2 μM) served as a control. The number of cells with an intact mitochondrial membrane potential is given in the upper right region (left). The ratio of cells with an intact mitochondrial membrane potential under normoxia/hypoxia is given for three independent experiments (right). **B.** The amount of apoptotic and necrotic cells was determined by annexin V/propidium iodide staining as recently described [35]. 1×10^4^ stained cells were analysed with a FACS Calibur (BD). The amount of vital cells after 24 h and 48 h cultivation is shown in the bar charts. Data represent the mean of three independent experiments. **C.** Cleaved caspase-3 activity was measured as recently described by Stehle and co-authors [35]. Both parental or CREB-deficient HER-2/neu^+^ cells were cultured under normoxic and hypoxic conditions before cleaved caspase-3 was determined as described in Materials and Methods using flow cytometry. **D.** ATP levels were measured with a luciferase specific substrate as described in Materials and Methods. Data show mean values from three independent experiments. **E.** Mitochondrial activity was analysed with the colorimetrical XTT substrate assay as described in Materials and Methods. Data show mean values from three independent experiments.

The hypoxia-induced partly translocation of CREB into mitochondria was accompanied by changes in the mitochondrial membrane potential of parental and CREB-deficient HER-2/neu^+^ cells. While the JC-1 fluorescence of normoxic parental and CREB-deficient HER-2/neu^+^ cells was comparable, hypoxia increased the mitochondrial membrane potential of parental HER-2/neu^+^ cells, but decreased it in CREB-deficient HER-2/neu^+^ cells (Figure 7A). The altered membrane potential during hypoxia directly correlated with changes in the apoptosis sensitivity: The viability of hypoxic CREB-deficient HER-2/neu^+^ cells were approximately 20% decreased when compared to hypoxic parental HER-2/neu^+^ cells (Figure 7B). This was further confirmed by an approximately 30% increased activity of caspase-3 (cleaved form) in normoxic CREB-deficient HER-2/neu^+^ cells, which was aggravated under hypoxia (Figure 7C). The cellular ATP levels were reduced in hypoxic parental and CREB deficient HER-2/neu^+^ cells, which were accompanied by a reduced mitochondrial activity. No changes were measured in the CREB deficient NIH3T3 cells between normoxic and hypoxic conditions. Furthermore hypoxia decreased ATP levels and mitochondrial activity in parental/CREB-deficient HER-2/neu^+^ cells (Figure 7D, 7E).

### Hypoxia-independent induction of cell migration and invasion by CREB activation

**Figure 8:**
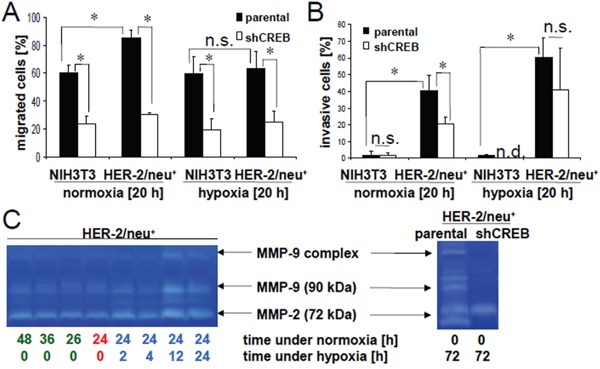
Abrogation of hypoxia-induced invasion by CREB silencing The influence of 20 h hypoxia or normoxia on the migration **A.** and invasion **B.** of HER-2/neu^−^ and HER-2/neu^+^ cells was analysed using trans-well inserts as described in Material and Methods. The bar charts represent three independent experiments performed in duplicates. **C.** The MMP activity under normoxic and hypoxic conditions in the culture supernatant was determined by gelatin zymography (left). Cells were incubated for 24 h under normoxic conditions (red) and were then incubated for the indicated time under hypoxia (blue) or were left under normoxia (green). The increased activity of MMP-9 and MMP-2 after 12 h or 24 h hypoxia is visible in both right lanes compared to the normoxic controls (left lanes). Parental and CREB-deficient HER-2/neu^+^ cells were incubated for 72 h under hypoxia and 20 μl supernatant was analysed on gelatin zymogram (right). A decreased activity of MMP-9 and of the cleaved MMP-2 was detected in CREB deficient cells, while the inactive MMP-2 (72 kDa) is not altered. The gels represent one of two independent experiments.

During prolonged hypoxia CREB expression and activation was induced thereby affecting the growth properties of cells [20]. Thus, the migration capacity of parental and CREB-deficient HER-2/neu^−^ and HER-2/neu^+^ cells was compared under normoxia and hypoxia. As shown in Figure 8A, the migration rate of normoxic HER-2/neu^−^ and HER-2/neu^+^ cells was approximately 60 to 70% reduced upon CREB silencing (36). Similar results were obtained in the presence of hypoxia demonstrating a reduced migration rate of CREB-deficient HER-2/neu^−^ and HER-2/neu^+^ cells of approximately 60%. A significantly increased invasion capacity of normoxic parental HER-2/neu^+^ cells compared to normoxic HER-2/neu^−^ cells was found using transwell assays with matrigel-coated wells. Under these conditions the invasion was 50% inhibited by CREB silencing of HER-2/neu^+^ cells, while an enhanced invasion was detected in hypoxic parental HER-2/neu^+^ (Figure 8B). Here the invasion capacity of CREB silenced HER-2/neu^+^ was highly variable, contrary to the unchanged invasion in the NIH3T3 shCREB cells. This was accompanied by an induction of MMPs in hypoxic parental versus CREB-deficient HER-2/neu^+^ cells as determined by zymography (Figure 8C) suggesting that the hypoxia-mediated induction of CREB activity affected the regulation of basal MMP expression. MMP9 secretion was further increased under hypoxic but not under normoxic conditions.

## DISCUSSION

CREB has been demonstrated to play a key role in the initiation as well as progression of tumors, since it modulates the expression of genes/proteins involved in cell proliferation, apoptosis, invasion and migration [23–26]. This was confirmed by high levels of CREB expression and activation in tumors of distinct origin, which often correlated with disease progression and poor patients' survival [24, 29, 30], as well as in *in vitro* models of ras and HER-2/neu transformation [35, 39]. Furthermore, recent studies suggest that CREB activation is also involved in prolonged/late hypoxic responses [20]. Since the cellular localization and kinetic of phosphorylation as well as other PTMs of CREB are important features for its pathophysiologic function in tumors it is essential to analyse the changes of the CREB status under normal and hypoxic conditions.

*In vitro* analysis demonstrated a hypoxia-induced time-dependent CREB phosphorylation at position Ser^133^, but not at position Ser^121^. An altered CREB phosphorylation at Ser^133^ was already detected one hour after hypoxia onset. During the acute phase of hypoxia CREB ubiquitination occurred in parental HER-2/neu^+^ cells, which was transient and dependent on CREB phosphorylation and could be reverted by treatment with the proteasome inhibitor MG-132. Thus, ubiquitination of CREB might be responsible for signal transduction during early phase of hypoxia in parental HER-2/neu^+^ cells, whereas CREB phosphorylation is responsible for both early and prolonged hypoxia. These results are in contrast to those reported by Nakayama [20] and might be explained by differences in the cell systems analysed. Furthermore, SUMOylation has been shown to regulate the activity and function of CREB under physiologic conditions [14–16]. These data were extended in this study to hypoxia-induced CREB SUMOylation, but its role during hypoxia requires further investigation.

The hypoxia-mediated enhanced CREB activation was directly associated with an upregulation of hypoxia-regulated molecules, like VEGF and ICAM-1. Furthermore, hypoxia caused a partly translocation of CREB from the nucleus to the mitochondrial matrix in parental HER-2/neu^+^ cells, which was accompanied by an induction of pERK, but not of pAKT and increased levels of pCREB^Ser133^. Although MMP expression and activity was induced by hypoxia in parental HER-2/neu^+^ cells and an altered CREB-dependent migration and invasion capacity of parental versus CREB-deficient HER-2/neu^+^ exists, hypoxic conditions did not or only marginally alter their growth properties. These data suggest that the hypoxia-dependent induction of MMPs was not linked to enhanced invasion and migration, probably because of the morphological changes of the HER-2/neu^+^ cells under hypoxia. However, these effects might alter the tumor microenvironment, which interfere with anti-tumoral immune responses by inhibiting T cell activation and cytotoxicity as well as by reducing antigen processing of dendritic cells [40].

**Figure 9:**
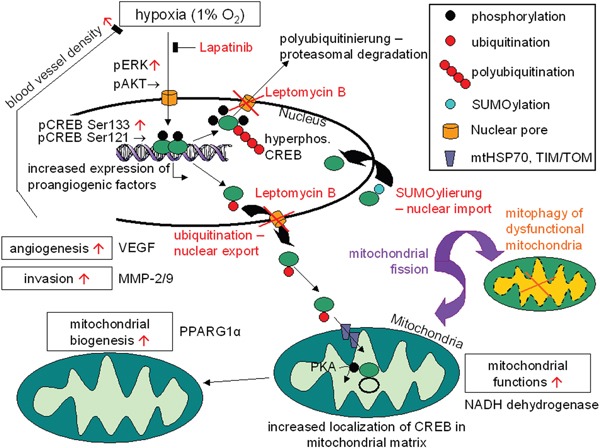
Model for the regulation and functions of CREB activity under hypoxic conditions in nucleus and mitochondria Hypoxia increases the activity of CREB (phosphorylation at Ser133 but not Ser121) by the MEK-ERK pathway, leading to an increased expression of pro-angiogenic factors (VEGF). This mechanism can counteract hypoxia. Hyperphosphorylation of CREB under hypoxia causes protein degradation by ubiquitination, while CREB can be stabilizied by SUMOylation. Import of modified CREB into mitochondria is enhanced under decreased oxygen supply, which in return can promote mitochondrial biogenesis and mitochondrial functions by binding to the mitochondrial promoter (D-LOOP) as well as regulating the expression of Ppargc1a.

A direct effect of CREB activation on angiogenesis was found, as determined by the increased number and density of blood vessels, CD31 staining of tumor lesions and enhanced expression of markers involved in angiogenesis. This could lead to a better oxygen and nutrient supply of parental HER-2/neu^+^ tumor cells. Furthermore, hypoxia did not only alter expression pattern of genes and proteins involved in neoplastic transformation, but could also affect the cellular localization of CREB. Although CREB is a nuclear transcription factor known to regulate gene expression, there exists some evidence that CREB could be co-localized to other cellular compartments. A hypoxia-induced mitochondrial translocation was found in parental HER-2/neu^+^ cells as determined by Western blot analysis of subcellular fractions, immunocytochemistry and analysis of mitochondrial gene expression (Figure 9). In the presence of hypoxia the pCREB^Ser133^ expression was significantly higher in the mitochondria than in the nucleus demonstrating the importance of the localization of CREB for its activity (mitochondrion = nucleus > cytosol). This was in line with a decreased expression of a subset of mitochondrial genes including ND5, COXIII and ATP synthase 6 upon disruption of CREB activity demonstrating that CREB is transcriptionally active in the mitochondria. Treatment of cells with leptomycin B caused an inhibition of mito-CREB. Since LY294002 had comparable effects, the AKT-mediated phosphorylation might be essential for the export of CREB into the mitochondria.

Mito-CREB has been shown to be involved in pathophysiologic processes of neurons and associated with altered apoptosis resistance [13]. This was correlated with changes in the mitochondrial membrane integrity and activation of caspases. The mitochondrial membrane potential provides a method for determination of early events of apoptosis. Indeed, hypoxic stress not only altered localization of CREB in parental HER-2/neu^+^ cells, but also the sensitivity to apoptosis. Furthermore, mitochondrial dysfunction can be caused by defects in the energy metabolism and by stress including oxidative damage or hypoxia. This might be involved in the pathogenesis of neoplastic diseases. The localization of CREB in the mitochondrion after hypoxia suggests that CREB mediates survival by regulating mitochondrial function and biogenesis thereby altering the response to various stimuli. In parental HER-2/neu^+^ cells the mitochondria were functional and increased respiratory rates correlated with the mitochondrial CREB levels. Increasing evidence suggests a close interrelationship between CREB overexpression and mitochondrial biogenesis in the presence of hypoxia. The adaption to hypoxia increased the biogenesis of novel and functional mitochondria. Similar results have been recently also demonstrated during the adaption to oxidative stress [41].

HER-2/neu signalling induced significant changes in the CREB status, in particular of CREB phosphorylation. While CREB activation occurs in response to pro-growth and pro-survival stimuli, it is also phosphorylated in response to harmful or stress stimuli, such as UV irradiation and hypoxia [42], due to an upregulation of the mitogen- and stress-activated protein kinase-1 [43]. Indeed, an increased activity of total and phosphorylated CREB along with a decreased localization of pCREB in the nucleus was demonstrated under hypoxia in parental HER-2/neu^+^ cells. pCREB^Ser133^, but not pCREB^Ser121^ appears to play an important role in this process, which is in line with recent reports suggesting that despite the existence of several phosphorylation sites, phosphorylation of CREB at Ser^133^ is the major modulator of CREB activity [44]. It cannot be ruled out, that the CREB-dependent gene expression may involve modifications of CREB at sites other than Ser^133^.

In conclusion our data support a shuttling pathway of HER-2/neu-mediated CREB activation linking CREB signalling to mitochondrial and nuclear functions. Furthermore, a hypoxia-mediated induction of CREB expression and altered PTMs followed by enhanced angiogenesis was observed. Increased knowledge of the role of HER-2/neu-induced CREB expression and activity might lead to the development of treatment strategies including inhibition of angiogenesis and alteration of the cellular metabolism.

## MATERIALS AND METHODS

### Cell culture, hypoxia and drug treatment

The generation and culture conditions of HER-2/neu^−^ NIH3T3 cells, their HER-2/neu^+^ derivative and their respective CREB variants have been recently described [35]. HER-2/neu-overexpressing cells with high levels of pCREB were termed “parental HER-2/neu^+^” cells, while HER-2/neu^+^ cells with silenced CREB were “HER-2/neu^+^ shCREB” cells. Human umbilical vein endothelial cells (HUVEC) purchased from the American Tissue Culture Collection (ATCC) were cultured in Ham's F-12K (ATCC) supplemented with 2% FBS, 1 ng/ml human epidermal growth factor (hEGF), 1 ng/ml human basic fibroblast growth factor (hbFGF), 1 μg/ml hydrocortison and 0.004 mg/ml ECGS (all supplements from PromoCell). The human mammary carcinoma cell lines HTB-122 (BT-549) and SKBR3, the lung cancer cell line H125 (NCI-H125) and the HER-2/neu-transformed cells have been previously described [45] and were cultivated in RPMI1640 supplemented with 10% FBS, 2 mM L-glutamine, 10 mM HEPES and penicillin/streptomycin. Cell lines were validated with their HER-2/neu^+^ and CREB expression status (murine cells) or with DNA fingerprinting (human cells).

For hypoxia treatment cells were incubated in a 1% (v/v) O_2_, 5% (v/v) CO_2_ atmosphere by 37°C for the indicated time. Normoxic controls were exposed using standard conditions. For Western blot and qPCR cells were cultured under normoxic and hypoxic conditions for up to 5 days and cell pellets were frozen until use. The proteasome inhibitor MG-132 (Calbiochem), the ubiquitin inhibitor PYR-41 (Santa Cruz) and the nuclear export inhibitor Leptomycin B (Cayman) were used with the indicated concentrations for 4 h (MG-132, PYR-41) or 24 h (Leptomycin B), respectively.

### RNA isolation, cDNA synthesis, qPCR

Total cellular RNA from 5×10^5^ cells/sample was extracted and subjected to qPCR analysis as recently described [35]. The specific primer sequences and PCR conditions used are given in Table 2A and 2B. Relative expression levels were calculated according to the ΔCt method and normalized against β-actin and/or GAPDH as internal control.

**Table 2:** Primer used for real time quantitative PCR

**A: Nuclear encoded genes**		
**Primer**	**Sequence**	**Annealing temperature [°C]**
GAPDH fwd	TTGTGCAGTGCCAGCCTCGT	60
GAPDH rev	TCGGCCTTGACTGTGCCGTT	60
CREB-1 fwd	CATTGCCCCTGGAGTTGTTATG	60
CREB-1 rev	TCTACGACATTCTCTTGCTGCCTC	60
Angiopoietin-1 fwd	TTGGGTCCAGAGAATGCCAC	60
Angiopoietin-1 rev	CCAGGTCACCTCCACAATCC	60
Angiopoietin-2 fwd	TTCTCCTCTGCCACGTTCAC	60
Angiopoietin-2 rev	TGTTGGCCCTGTCGACATTT	60
Beta-actin fwd	CTGTCGAGTCGCGTCCACCC	60
Beta-actin rev	TGCTCTGGGCCTCGTCACCC	60
ICAM-1 fwd	TGTCAGCCACCATGCCTTAG	60
ICAM-1 rev	CAGCTTGCACGACCCTTCTA	60
PECAM-1 fwd	CCAAGGCCAAACAGAAACCC	60
PECAM-1 rev	GTTCTTAGGGTCGACCTTCCG	60
VCAM-1 fwd	GTACAGTCTGGTGGAGGCAC	60
VCAM-1 rev	GCATGGCTTGGTTTGTGGAG	60
VEGF A fwd	AACCATGAACTTTCTGCTGTCTTGG	60
VEGF A rev	ATCAGGGTACTCCTGGAAGATGTCC	60
PPARGC1a fwd	CAAAGCAGCAGAGAGGGAAC	60
PPARGC1a rev	CTTCGTACAGCCATCAAAAAGGG	60
18S rDNA fwd	TAGAGGGACAAGTGGCGTTC	60
18S rDNA rev	CGCTGAGCCAGTCAGTGT	60
**B: Mitochondrial encoded genes**		
**Primer**	**Sequence**	**Annealing temperature [°C]**
mATPSyn6 fwd	CCAATGGCATTAGCAGTCCG	60
mATPSyn6 rev	TGCATGAGTTTGGTGGGTCA	60
mATPSyn8 fwd	CAAACATTCCCACTGGCACC	60
mATPSyn8 rev	TTGTTGGGGTAATGAATGAGGC	60
mCOX I fwd	GACTTGCAACCCTACACGGA	60
mCOX I rev	GATGGCGAAGTGGGCTTTTG	60
mCOX II fwd	AACCGAGTCGTTCTGCCAAT	60
mCOX II rev	CTAGGGAGGGGACTGCTCAT	60
mCOX III fwd	CCAATGGCATTAGCAGTCCG	60
mCOX III rev	TGCATGAGTTTGGTGGGTCA	60
mND1 fwd	TCCGAGCATCTTATCCACGC	60
mND1 rev	GTATGGTGGTACTCCCGCTG	60
mND2 fwd	CAAGGGATCCCACTGCACAT	60
mND2 rev	AAGTCCTCCTCATGCCCCTA	60
mND3 fwd	GTTGCATTCTGACTCCCCCA	60
mND3 rev	GGTAGACGTGCAGAGCTTGT	60
mND4 fwd	AGCTCCAATTGCTGGGTCAA	60
mND4 rev	GGCTAACTGAGGAGTAGGCG	60
mND4L fwd	ACCTCACCATAGCCTTCTCAC	60
mND4L rev	TAGTCCTACAGCTGCTTCGC	60
mND5 fwd	ACCCAATCAAACGCCTAGCA	60
mND5 rev	AGGACTGGAATGCTGGTTGG	60
mND6 fwd	GAAGGAGGGATTGGGGTAGC	60
mND6 rev	CCGCAAACAAAGATCACCCAG	60
COXI fwd	GCCCCAGATATAGCATTCCC	60
COXI rev	GTTCATCCTGTTCCTGCTCC	60
**C: Primer used for real time quantitative PCR after mito DNA IP**		
**Primer**	**Sequence**	**Annealing temperature [°C]**
Mito D-LOOP 1	GTGGTGTCATGCATTTGGTATCT	60
Mito D-LOOP 2	ATCAACATAGCCGTCAAGGCATG	60
Mito D-LOOP 3	TCACCGTAGGTGCGTCTAGACTGT	60

### Protein isolation, cell fractionation and western blot analysis

From 5×10^6^ cells/sample protein was isolated and subjected to Western blot analysis as recently described [35] using following monoclonal antibodies (mAb): anti-phospho-CREB (Ser^133^), anti-phospho-CREB (Ser^121^) (Novus), anti-GAPDH, β-actin (Sigma), anti-phospho-ERK, anti-ERK, anti-phospho-AKT, anti-AKT and anti-histone-3. More detailed information regarding the antibodies used in this study are given in Table 3.

**Table 3:** Primary and labeled secondary antibodies used in this study

Antigen	Species	Manufacturer (#)	monoclonal	Application	Dilution	Dilution buffer
CREB-1	Rabbit	Cell Signaling (#9197)	X (48H2)	Western Blot	1:1000	5 % (w/v) BSA in TBS-T
				ICC	1:800	HBSS + 0,1 % Triton X100
				IHC	1:400	Signal Stain Antibody Diluent (Cell Signaling)
				ChIP	1:100	ChIP1 buffer
				IP	1:250	IP buffer
CREB-1	Mouse	Cell signaling (#9104)	X (86B10)	Western Blot (after IP)	1:1000	5 % (w/v) BSA in TBS-T
phospho-CREB-1 (Ser133)	Rabbit	Cell Signaling (#9198)	X (87G3)	Western Blot	1:1000	5 % (w/v) BSA in TBS-T
phospho-CREB-1 (Ser121)	Rabbit	Novus (#NB100-410)	polyclonal	Western Blot	1:1000	5 % (w/v) BSA in TBS-T
anti-mouse IgG-HRP	Horse	Cell Signaling (#7076)	n/a	Western Blot	1:5000	5 % (w/v) Skim milk in TBS-T
anti-rabbit IgG-HRP	Goat	Cell Signaling (#7074)	n/a	Western Blot	1:5000	5 % (w/v) Skim milk in TBS-T
AIF1	Rabbit	Cell Signaling (#5318)	X (D39D2)	Western Blot	1:1000	5 % (w/v) BSA in TBS-T
AKT	Rabbit	Cell Signaling (#9272)	polyclonal	Western Blot	1:2000	5 % (w/v) BSA in TBS-T
phospho-AKT (Ser473)	Rabbit	Cell Signaling (#9271)	polyclonal	Western Blot	1:1000	5 % (w/v) BSA in TBS-T
*β*-Actin	Mouse	Sigma (#A2228)	X (AC74)	Western Blot	1:5000	5 % (w/v) Skim milk in TBS-T
CD31	Rabbit	Abbiotech (#250590)	n/a	IHC	1:200	PBS-T, 5 % goat serum
COX IV	Rabbit	Novus (#NB110-39115)	polyclonal	Western Blot	1:2000	5 % (w/v) BSA in TBS-T
ERK-1/2	Rabbit	Cell Signaling (#9102)	polyclonal	Western Blot	1:2000	5 % (w/v) BSA in TBS-T
phospho-ERK1/2 (Thr202/204)	Rabbit	Cell Signaling (#4370)	X (D1314.4E)	Western Blot	1:2000	5 % (w/v) BSA in TBS-T
GAPDH	Rabbit	Cell Signaling (#2118)	X (14C10)	Western Blot	1:2000	5 % (w/v) BSA in TBS-T
HER-2/neu-PE (IgG1)	Mouse (IgG1)	BD (#340552)	X (neu 24.7)	Flow cytometry	1:200	PBS
HIF 1-α	Rabbit	Biorbyt (#orb223706)	X (n/a)	IHC	1:200	PBS-T, 5 % goat serum
Histone H3	Mouse	Cell Signaling (#3638)	X (96C10)	Western Blot	1:1000	5 % (w/v) BSA in TBS-T
IgG1-PE	Mouse	BC (#A07796)	X (679.1Mc7)	Flow cytometry	1:200	PBS
Ki-67	Rabbit	Novus (#NB110-89717)	polyclonal	IHC	1:250	PBS-T, 5 % goat serum
MEK1	Mouse	Cell Signaling (#2352)	X (61B12)	Western Blot	1:2000	5 % (w/v) Skim milk in TBS-T
PDK4	Rabbit	Biorbyt (#orb11259)	polyclonal	Western Blot	1:1000	5 % (w/v) BSA in TBS-T
SUMO-1	Rabbit	Cell Signaling (#4930)	polyclonal	Western Blot	1:1000	5 % (w/v) BSA in TBS-T
				IP	1:50	IP buffer
TOM20	Rabbit	Biorbyt (#orb128858)	polyclonal	Western Blot	1:1000	5 % (w/v) BSA in TBS-T
*β*-Tubulin	Mouse	Oncogene	X (Ab-1)	Western Blot	1:2000	5 % (w/v) Skim milk in TBS-T
Ubiquitin	Mouse	Millipore (#MAB1510)	X (Ubi1)	Western Blot	1:1000	5 % (w/v) BSA in TBS-T

The mouse CREB-1 antibody was used for the Western-Blot after IP.

Furthermore, cells were fractionated into cytosolic, mitochondrial and nuclear compartments using a cell fractionation kit (Cell Signaling) according to the manufacturer's instructions. The isolation of subcellular compartments allows the analysis of the distribution of CREB in the cytosol, mitochondria and nucleus. The purity of the subcellular fractions was determined using histone H3 as nuclear, AIF as mitochondrial and MEK as cytosolic marker.

### Ubiquitin pull down assay, CREB immunoprecipitation and ubiquitin quantification

Cells were cultivated for 24 h under normoxia or hypoxia, harvested and lysed in 50 mM HEPES (pH 7.5), 5 mM EDTA, 50 mM NaCl, 1% Triton X-100, 10 mM N-ethylmaleimide and a protease inhibitor cocktail. After centrifugation, ubiquitin affinity beads (Ubiquitinated Protein Enrichment Kit, Calbiochem) were added to the supernatant with a protein concentration of ~1 mg/ml and pull down assays were performed according to the manufacturer's protocol. The amount of ubiquitinated CREB protein was detected by Western blot using an anti-ubiquitin (Millipore) and anti-CREB-specific antibody (Cell Signaling).

For quantification, cell lysates were incubated with an anti-CREB antibody (Cell Signaling) overnight under agitation at 4°C, before 20 μl protein A agarose was added for 4 hrs. Beads were collected by centrifugation, washed four times with 200 μl wash buffer (50 mM HEPES pH 7.5, 5 mM EDTA, 50 mM NaCl) and heated in 50 μl Laemmli buffer. The supernatant was loaded onto a 12% SDS gel following Western Blot analysis with an ubiquitin-specific antibody.

### SUMO detection

Cell lysates were incubated over night by 4°C under slight agitation with a monoclonal SUMO-1 antibody (Cell Signaling #4930, 1:50 dilution). Protein A agarose slurry was added and was incubated for 2 h at 4°C (Sigma). After a centrifugation step (16.000 *g*, 30 s) the agarose beats were collected, washed four times with 200 μl wash buffer and were heated in laemmli buffer for 5 min. The protein extract was separated on a 10% SDS gel.

### Migration and invasion assays

For determination of cell migration 1×10^5^ cells cultured in medium supplemented with 1% FBS were seeded into the upper well of the chamber, whereas medium containing 10% FCS was added to the lower chamber of the trans-well chamber system (Corning). After incubation for 18 h at 37°C, non-migrated cells of the top insert were completely removed, whereas the cells migrated to the lower chamber were lysed with Cell Titer Glo (Promega) before the ATP content was determined according to the manufacturer's instructions using a luminometer (Berthold).

For invasion assays trans-well chambers were coated with 50 μl matrigel (Sigma) prior the experiments. Each experiment was performed in triplicates at least three times.

### Analysis of CREB binding to mtDNA

Proteins and mtDNA of hypoxic or normoxic conditioned cells were cross-linked by 1% formaldehyde for 5 min following the incubation with 1M glycine for 5 min. The cells were lysed and sonicated three times. After centrifugation, 10 μg of the shredded protein-DNA was incubated with the CREB antibody or a rabbit IgG control antibody over night for immunoprecipitation. Treatment with agarose A/G beads for 2 h and centrifugation collected the specific DNA, which was eluted by adding 1% SDS, 0.1 M NaHCO_3_ pH 8.0 buffer. The crosslink was reverted by incubation for 4 h at 65°C and proteins were digested by protease K. The DNA was purified by phenol-chloroform extraction and ethanol cleaning. mtDNA was detected as described by Ryu and co-workers [13] using the primers in Table 2C.

### *In vivo* tumorigenicity

For all animal experiments mice were housed under standard conditions as described previously and the study was proofed by the ethical committee [35]. Briefly, 1×10^6^ cells/mouse were *subcutaneously* (s.c.) injected into the flank of DBA-1 mice (2 - 3 month old, male and female). The tumor growth and size were monitored overtime. Mice were killed 4 - 5 weeks after injection before tumors were isolated.

### *In vivo* angiogenesis assays

The *in vivo* angiogenesis assay was performed by injecting 1×10^6^ cells/mouse suspended in 250 or 500 μl growth factor reduced matrigel into the flank of DBA-1 mice. Seven days after injection the mice were sacrificed and plugs were removed. The plugs were divided and one half was embedded after formalin fixation into paraffin for histological staining, while the other part was used for hemoglobin measurement.

### Determination of the hemoglobin content

The matrigel plugs were homogenised with an ultra turrax homogenizer in 100 μl erythrocyte lysis buffer and the hemoglobin concentration was measured in the supernatant after centrifugation using the cyanomethemoglobin method. Briefly, 100 μl sample or blank were mixed with 25 μl Drabkin's reagent and after 10 min the absorption was measured at 540 nm. Control blood was used as a standard.

### *In vitro* angiogenesis assays

For determination of *in vitro* angiogenesis 5×10^3^ HUVEC/well were seeded into flat bottom 96 well plates onto polymerized matrigel and 100 μl conditioned cell culture supernatant obtained from parental or CREB-deficient HER-2/neu^+^ cells was added. The cells were incubated for 16 h before the cell morphology and branches was analysed under a microscope.

### Histology, immunohistochemistry (IHC) and immunocytochemistry (ICC)

Tumors or matrigel plugs were fixed in 5% (v/v) formaldehyde overnight and then embedded in paraffin. Tumor samples were cut into 5 μm slices and then incubated with primary mAb directed against CD31 (Abbiotec) or HIF-1α (Novus) for 24 h. A compatible secondary antibody linked with HRP SignalStain^®^ Boost IHC Detection Reagent (Cell Signaling) was used for the detection with the substrate DAB. Blood vessels of anti-CD31-stained slides were counted and their mean number calculated to determine the blood vessel density.

Matrigel plug slides were stained with hematoxyline-eosine, Giemsa or Masson-Goldner [46].

Immunocytochemical analysis was performed with 5×10^3^ cells/well seeded into clear bottom black 96 well plates (BD) and incubated in DMEM without phenol red for 24 h by 37°C. Cells were washed with PBS and fixed with 4% paraformaldehyde for 20 min. After washing with HBSS and 0.5% Triton X-100 (HBSS-T) cells were permeabilized in HBSS-T for 20 min, then incubated over night at 4°C with the primary antibody (CREB in HBSS-T) followed by washing with HBSS-T three times. After incubation for 1 h with a secondary Alexa488-linked anti-rabbit polyclonal antibody (Cell Signaling) at 4°C, the cells were washed with HBSS-T following HBSS. DAPI was employed for the nuclear staining and fluorescence was analysed with a fluorescence microscope (Leica).

For evaluation of localization of CREB in human breast cancer tissue, formalin-fixed paraffin embedded tissue samples that contained necrotic areas indicative of localized hypoxia were stained with the phospho-CREB (Ser133) (87G3) Rabbit mAb (Cell Signaling) in the indicated concentration using the Ventana Discovery Autostainer (Ventana).

### Determination of the MMP activity by zymography

Gelatin degradation activity of MMP-2 and 9 in the culture supernatant was determined with the gelatin zymography as recently described [35].

### Immunofluorescence microscopy of mitochondria

Mitochondria were visualized using the MitoTracker Red CMXROS (Life Technologies) and DAPI (Calbiochem) was added for nuclear staining. Cells were immediately analysed using a fluorescence microscope at a 20-fold magnification.

### Flow cytometry

Cells were harvested after 24 h under normoxia or hypoxia and stained with an anti-HER-2/neu-PE (BD) antibody or with the IgG1-PE isotype control (BD), respectively, for 20 min as recently described [47]. Fluorescence intensity was determined by flow cytometry (FACSCalibur, BD). The results were expressed as mean specific fluorescence intensity (MFI). Three independent experiments were performed.

### Apoptosis assays and determination of the mitochondrial membrane potential

Apoptosis was determined as previously described using annexin V and caspase-3 staining, respectively [35]. For determination of the mitochondrial membrane potential as an indicator for apoptosis cells were treated for 24 h under normoxia or hypoxia, before harvesting by trypsination. After centrifugation cells were stained with 2 μM JC-1 (Invitrogen) for 30 min at 37°C and fluorescence was measured with a FACSCalibur.

### Determination of the mitochondrial activity and ATP content

5×10^3^ cells per 96 well were incubated for 24 h and mitochondrial activity was determined by using the XTT viability assay kit (Roche) according to the manufacturer's instructions. ATP levels of normoxic and hypoxic cells were analysed with the CellTiterGlo reagent (Promega). Values were normalized to cells cultured under normoxic condition.

### Analysis of cAMP concentrations

The concentration of cAMP in the cells was determined with the cAMP-Glo Assay kit (Promega). Briefly, 5×10^3^ cells/well were seeded into a white clear-bottom plate (BD) and then cultivated for 24 h under normoxia or hypoxia. The measurement of the cAMP was performed as described in the manufacturer's instructions. Luminescence in the plates were analysed with a luminometer. The experiments were performed trice.

### Extracellular flux assays

Bioenergetics of parental and CREB-deficient HER-2/neu^+^ and in HER-2/neu^−^ cells were determined using the XF96e Extracellular Flux analyser (Seahorse Biosience) as recently described [41]. Briefly, 2×10^5^ cells/well were reseeded in specialized tissue culture plates (96FX micro well plate). One hour prior measurement, cells were incubated at 37°C in a CO_2_-free atmosphere. First basal oxygen consumption rate (OCR), which is an indicator for mitochondrial respiration was detected. In the next step OCR responses toward the application of 1 μM oligomycin, 2.5 μM FCCP (carbonyl cyanide-p-trifluoromethyloxy-phenylhydrazane) and the combination of 3 μM antimycin and 3 μM rotenone (XF Cell Mito Stress Test Kit, Seahorse Bioscience) were evaluated. All experiments were performed in at least octaplicates.

### Determination of mitochondrial DNA copy number, mitochondrial mass and oxidative potential

Genomic and mitochondrial DNA (mtDNA) was isolated from normoxic and hypoxic HER-2/neu^+^ cells using the QiaAMP DNA mini kit following the manufacturer's instructions. The copy number of mtDNA was determined by qPCR as described [48]. For determination of the mitochondrial mass and oxidative potential cells were stained with 100 nM of the mitochondrial-specific dye MitoTracker Green (Life Technologies) according to the manufacturer's instructions for staining the mitochondrial lipid membrane and with 100 nM MitoTracker Red for determination of oxidation for 20 min at 37°C followed by flow cytometry [41].

### Mitochondrial sub fractionation and determination of mitochondrial complex activities

Cells were harvested and washed with PBS two times. After resuspension in 225 mM sorbitol, 75 mM sucrose, 0.1 mM EGTA and 30 mM Tris HCl pH 7,4 cells were ultrasonified (3 × 5 strokes with 10% frequence intensity). Upon centrifugation of lysate (600 *g*, 5 min, 4°C, repeated two times) the supernatant was centrifuged by 7000 *g* for 10 min at 4°C. The mitochondrial crude pellet was dissolved in 200 μl 10 μM KH_2_PO_4_ and was incubated for 20 min on ice. 200 μl isotonic buffer (32% sucrose, 30% glycerol, 10 mM MgCl_2_) were added prior to centrifugation at 10,000 *g* for 10 min at 4°C.

The supernatant representing the outer membrane and the protein of the inter membrane space of mitochondrial was centrifuged for 1 h at 4°C and 15,000 *g*, resulting in a pellet P2 with the inner membrane proteins, while the supernatant S2 contains the proteins of the inter membrane space. The pellet representing the inner membrane and the matrix was resuspended in 200 μl 10 μM KH_2_PO_4_ pH 8.0 and followed by incubation for 20 min on ice. 200 μl isotonic buffer (32% sucrose, 30% glycerol, 10 mM MgCl_2_) were added and the mixture was centrifuged (15,000 *g*, 1 h, 4°C). Pellet P3 are the proteins in the inner membrane while the supernatant S3 contains the mitochondrial matrix proteins. The four fractions as well as the crude extract were separated on a 12% SDS PAGE and proteins were identified by Western Blot analysis.

For determination of mitochondrial complex activities, crude mitochondria extracts were solubilized by adding DDM reaching a concentration of 0.45%. After centrifugation the supernatant was used for the activity measurement.

Complex I activity was determined by adding 1 ml NADH buffer (100 μM NADH, 10 mM KH_2_PO_4_ pH 8.0) to 50 μg mitochondrial proteins following the absorption at 340 nm for 1 min. After addition of 5 μl 10 mM ubichinon-10 the absorption was measured for 2 min. The turn-over rate was calculated with ε NADH = 6.21 mM^−1^*cm^−1^.

Complex II activity was measured by adding 25 μg mitochondrial proteins to 1 ml reaction buffer (50 mM KH_2_PO_4_ (pH 7.5), 90 μM ubichinon-2, 55 μM dichlorophenolindophenol and 10 mM sodium succinate).

### Blue native gel electrophoresis and in gel activity staining

Mitochondria (~ 1 mg) were prepared in ACA buffer (500 mM 6-aminocaproic acid, 0.5 mM EDTA, 50 mM BisTris (pH 7.0)) and were solubilized by adding DDM (concentration: 0.45%). Samples were gentle mixed at 4°C for 30 min and were centrifuged for 30 min at 4°C and 16,000 *g*. 200 μg mitochondrial proteins in the supernatant were mixed with blue native loading buffer leading to an end concentration of 10% glycerol, 0.05% Serva Blue G250 and 500 mM 6-aminocaproic acid. The sample were loaded onto 4-16% native gradient gels and protein complexes were separated by 45 V for 19 - 24 h under 4°C cooling using 50 mM BisTris pH 7.0 as the anode buffer and 50 mM Tricine, 15 mM BisTris, 0.002% Serva Blue G250 as cathode buffer. All components were from the ServaGel Native Gel starter kit (Serva). In gel activity staining was performed as described by [49].

### Statistical analysis and Kaplan-Meier survival analysis

Differences between groups were analysed by Mann-Whitney test, Student's t test or ANOVA as appropriate. Data are expressed as mean ± SD and p values reported are 2-sided. Significance were accepted if p values were ≤ 0.05, *, ≤ 0.01, **. For the Kaplan-Meier curve analysis the KMplot database was used with the Affymetrix ID 225572_at for CREB1 and 200989_at for HIF-1α (http://kmplot.com/analysis/index.php?p=service&cancer=breast). The chosen Affymetrix IDs had the best JetSet score [50]. The following settings were used for the data analysis: Split patients by: median, Follow up threshold: all, Censore at threshold: checked, Compute median over entire database: false. Cut-off values and the expression range of the samples were automatically adjusted by the database and are depicted in the survival curves.

## SUPPLEMENTARY FIGURES AND TABLE



## Abbreviations

AIF: Apoptosis-inducing factor
AKT, ATCC: American Tissue Culture Collection
CaMK: calcium activated calmodulin kinase
CBP: CREB binding protein
CCCP: carbonyl cyanide *m*-chlorophenyl hydrazone
CD: cluster of differentiation
COX: cytochrome c oxidase
CRE: cAMP-responsive element
CREB: cAMP-responsive element-binding protein
ERK: extracellular-signal regulated kinase
FCCP: Carbonyl cyanide-*4*-(trifluoromethoxy)phenylhydrazone
DAPI: 4′,6-diamidino-2-phenylindole
DDM: n-Dodecyl β-D-maltoside
FGF: fibroblast growth factor
GAPDH: Glyceraldehyde 3-phosphate dehydrogenase
GLUT 1: glucose transporter 1
GSH: glutathione
HBSS: Hank's buffered salt solution
hbFGF: human basic fibroblast growth factor
hEGF: human epidermal growth factor
HER-2/neu: human epidermal growth factor receptor 2 / neuroblastoma
HIF: hypoxia inducible factor
HRE: hypoxia responsible element
HUVEC: human umbilical vein endothelial cells
hypo: hypoxia
JC-1: 5,5′,6,6′-tetrachloro-1,1′,3,3′-tetraethylbenzimidazolylcarbocyanine iodide
mAb: monoclonal antibody
MAPK: mitogen-activated protein kinase
MEK: MAP/ERK kinase
MFI: mean fluorescence intensity
mito-CREB: mitochondrial CREB
MMP: matrix metalloproteinase
mtHSP70: mitochondrial heat shock protein 70 family
NADH: nicotinamide adenine dinucleotide
NC: non-sense control
ND: NADH dehydrogenase
NF: nuclear factor
OCR: oxygen consumption rate
OM: mitochondrial outer membrane
PDK4: pyruvate dehydrogenase kinase isoform 4
PE: phycoerythin
PECAM: Platelet endothelial cell adhesion molecule
PPARGc1a: Peroxisome proliferator-activated receptor gamma coactivator 1-alpha
PKA: protein kinase A
ProtK: proteinase K
PTM: post-transcriptional modification
qPCR: quantitative PCR
SUMO: small ubiquitin-related modifier
TOM20: translocase of outer membrane
VCAM: vascular cellular adhesion molecule
VEGF: vascular endothelial growth factor.

## References

[1] Dodson GE, Tibbetts RS (2006). DNA replication stress-induced phosphorylation of cyclic AMP response element-binding protein mediated by ATM. J Biol Chem.

[2] Johannessen M, Delghandi MP, Moens U (2004). What turns CREB on?. Cell Signal.

[3] Shi Y, Venkataraman SL, Dodson GE, Mabb AM, LeBlanc S, Tibbetts RS (2004). Direct regulation of CREB transcriptional activity by ATM in response to genotoxic stress. Proc Natl Acad Sci U S A.

[4] Ishimoto T, Mano H, Ozawa T, Mori H (2012). Measuring CREB activation using bioluminescent probes that detect KID-KIX interaction in living cells. Bioconjug Chem.

[5] Braeuer RR, Zigler M, Villares GJ, Dobroff AS, Bar-Eli M (2011). Transcriptional control of melanoma metastasis: the importance of the tumor microenvironment. Semin Cancer Biol.

[6] Cho EC, Mitton B, Sakamoto KM (2011). CREB and leukemogenesis. Crit Rev Oncog.

[7] De Rasmo D, Signorile A, Roca E, Papa S (2009). cAMP response element-binding protein (CREB) is imported into mitochondria and promotes protein synthesis. FEBS J.

[8] Kotla S, Singh NK, Heckle MR, Tigyi GJ, Rao GN (2013). The transcription factor CREB enhances interleukin-17A production and inflammation in a mouse model of atherosclerosis. Sci Signal.

[9] Persengiev SP, Green MR (2003). The role of ATF/CREB family members in cell growth, survival and apoptosis. Apoptosis.

[10] Bevilaqua LR, Cammarota M, Paratcha G, de Stein ML, Izquierdo I, Medina JH (1999). Experience-dependent increase in cAMP-responsive element binding protein in synaptic and nonsynaptic mitochondria of the rat hippocampus. Eur J Neurosci.

[11] Lee J, Sharma S, Kim J, Ferrante RJ, Ryu H (2008). Mitochondrial nuclear receptors and transcription factors: who's minding the cell?. J Neurosci Res.

[12] Ryu BJ, Lee H, Kim SH, Heo JN, Choi SW, Yeon JT, Lee J, Lee J, Cho JY, Kim SH, Lee SY (2014). PF-3758309, p21-activated kinase 4 inhibitor, suppresses migration and invasion of A549 human lung cancer cells via regulation of CREB, NF-kappaB, and beta-catenin signalings. Mol Cell Biochem.

[13] Ryu H, Lee J, Impey S, Ratan RR, Ferrante RJ (2005). Antioxidants modulate mitochondrial PKA and increase CREB binding to D-loop DNA of the mitochondrial genome in neurons. Proc Natl Acad Sci U S A.

[14] Chen YC, Hsu WL, Ma YL, Tai DJ, Lee EH (2014). CREB SUMOylation by the E3 Ligase PIAS1 Enhances Spatial Memory. J Neurosci.

[15] Comerford KM, Leonard MO, Karhausen J, Carey R, Colgan SP, Taylor CT (2003). Small ubiquitin-related modifier-1 modification mediates resolution of CREB-dependent responses to hypoxia. Proc Natl Acad Sci U S A.

[16] Ryan CM, Kindle KB, Collins HM, Heery DM (2010). SUMOylation regulates the nuclear mobility of CREB binding protein and its association with nuclear bodies in live cells. Biochem Biophys Res Commun.

[17] Seo SR, Chung KC (2008(a)). CREB activates proteasomal degradation of DSCR1/RCAN1. FEBS Lett.

[18] Melchior F (2000). SUMO—nonclassical ubiquitin. Annu Rev Cell Dev Biol.

[19] Taylor CT, Furuta GT, Synnestvedt K, Colgan SP (2000). Phosphorylation-dependent targeting of cAMP response element binding protein to the ubiquitin/proteasome pathway in hypoxia. Proc Natl Acad Sci U S A.

[20] Nakayama K (2013). cAMP-response element-binding protein (CREB) and NF-kappaB transcription factors are activated during prolonged hypoxia and cooperatively regulate the induction of matrix metalloproteinase MMP1. J Biol Chem.

[21] Cammarota M, Paratcha G, Bevilaqua LR, Levi de Stein M, Lopez M, Pellegrino de Iraldi A, Izquierdo I, Medina JH (1999). Cyclic AMP-responsive element binding protein in brain mitochondria. J Neurochem.

[22] Mootha VK, Bunkenborg J, Olsen JV, Hjerrild M, Wisniewski JR, Stahl E, Bolouri MS, Ray HN, Sihag S, Kamal M, Patterson N, Lander ES, Mann M (2003). Integrated analysis of protein composition, tissue diversity, and gene regulation in mouse mitochondria. Cell.

[23] Cheng JC, Esparza S, Sandoval S, Shankar D, Fu C, Sakamoto KM (2007). Potential role of CREB as a prognostic marker in acute myeloid leukemia. Future Oncol.

[24] Seo HS, Liu DD, Bekele BN, Kim MK, Pisters K, Lippman SM, Wistuba II, Koo JS (2008). Cyclic AMP response element-binding protein overexpression: a feature associated with negative prognosis in never smokers with non-small cell lung cancer. Cancer Res.

[25] Shankar DB, Cheng JC, Sakamoto KM (2005). Role of cyclic AMP response element binding protein in human leukemias. Cancer.

[26] Siu YT, Jin DY (2007). CREB—a real culprit in oncogenesis. FEBS J.

[27] Park JK, Park SH, So K, Bae IH, Yoo YD, Um HD (2010). ICAM-3 enhances the migratory and invasive potential of human non-small cell lung cancer cells by inducing MMP-2 and MMP-9 via Akt and CREB. Int J Oncol.

[28] Abramovitch R, Tavor E, Jacob-Hirsch J, Zeira E, Amariglio N, Pappo O, Rechavi G, Galun E, Honigman A (2004). A pivotal role of cyclic AMP-responsive element binding protein in tumor progression. Cancer Res.

[29] Deng X, Liu H, Huang J, Cheng L, Keller ET, Parsons SJ, Hu CD (2008). Ionizing radiation induces prostate cancer neuroendocrine differentiation through interplay of CREB and ATF2: implications for disease progression. Cancer Res.

[30] Pigazzi M, Manara E, Bresolin S, Tregnago C, Beghin A, Baron E, Giarin E, Cho EC, Masetti R, Rao DS, Sakamoto KM, Basso G (2013). MicroRNA-34b promoter hypermethylation induces CREB overexpression and contributes to myeloid transformation. Haematologica.

[31] Wang Z, Zhang L, Ni Z, Sun J, Gao H, Cheng Z, Xu J, Yin P (2015). Resveratrol induces AMPK-dependent MDR1 inhibition in colorectal cancer HCT116/L-OHP cells by preventing activation of NF-kappaB signaling and suppressing cAMP-responsive element transcriptional activity. Tumour Biol.

[32] Vaupel P, Mayer A, Briest S, Hockel M (2003). Oxygenation gain factor: a novel parameter characterizing the association between hemoglobin level and the oxygenation status of breast cancers. Cancer Res.

[33] Li D, Jin L, Alesi GN, Kim YM, Fan J, Seo JH, Wang D, Tucker M, Gu TL, Lee BH, Taunton J, Magliocca KR, Chen ZG, Shin DM, Khuri FR, Kang S (2013). The prometastatic ribosomal S6 kinase 2-cAMP response element-binding protein (RSK2-CREB) signaling pathway up-regulates the actin-binding protein fascin-1 to promote tumor metastasis. J Biol Chem.

[34] Wang H, Huang M, Zhang DY, Zhang F (2011). Global profiling of signaling networks: study of breast cancer stem cells and potential regulation. Oncologist.

[35] Steven A, Leisz S, Massa C, Iezzi M, Lattanzio R, Lamolinara A, Bukur J, Muller A, Hiebl B, Holzhausen HJ, Seliger B (2013). HER-2/neu mediates oncogenic transformation via altered CREB expression and function. Mol Cancer Res.

[36] Martinez-Outschoorn U, Sotgia F, Lisanti MP (2014). Tumor microenvironment and metabolic synergy in breast cancers: critical importance of mitochondrial fuels and function. Semin Oncol.

[37] Lee J, Kim CH, Simon DK, Aminova LR, Andreyev AY, Kushnareva YE, Murphy AN, Lonze BE, Kim KS, Ginty DD, Ferrante RJ, Ryu H, Ratan RR (2005). Mitochondrial cyclic AMP response element-binding protein (CREB) mediates mitochondrial gene expression and neuronal survival. J Biol Chem.

[38] Szczepanek K, Lesnefsky EJ, Larner AC (2012). Multi-tasking: nuclear transcription factors with novel roles in the mitochondria. Trends Cell Biol.

[39] Steven A, Heiduk M, Recktenwald CV, Hiebl B, Wickenhauser C, Massa C, Seliger B (2015). Colorectal Carcinogenesis: Connecting K-RAS-Induced Transformation and CREB Activity In Vitro and In Vivo. Mol Cancer Res.

[40] Singer K, Kastenberger M, Gottfried E, Hammerschmied CG, Buttner M, Aigner M, Seliger B, Walter B, Schlosser H, Hartmann A, Andreesen R, Mackensen A, Kreutz M (2011). Warburg phenotype in renal cell carcinoma: high expression of glucose-transporter 1 (GLUT-1) correlates with low CD8(+) T-cell infiltration in the tumor. Int J Cancer.

[41] Jitschin R, Hofmann AD, Bruns H, Giessl A, Bricks J, Berger J, Saul D, Eckart MJ, Mackensen A, Mougiakakos D (2014). Mitochondrial metabolism contributes to oxidative stress and reveals therapeutic targets in chronic lymphocytic leukemia. Blood.

[42] Yao K, Chen H, Liu K, Langfald A, Yang G, Zhang Y, Yu DH, Kim MO, Lee MH, Li H, Bae KB, Kim HG, Ma WY, Bode AM, Dong Z, Dong Z (2014). Kaempferol targets RSK2 and MSK1 to suppress UV radiation-induced skin cancer. Cancer prevention research.

[43] Deak M, Clifton AD, Lucocq LM, Alessi DR (1998). Mitogen- and stress-activated protein kinase-1 (MSK1) is directly activated by MAPK and SAPK2/p38, and may mediate activation of CREB. The EMBO journal.

[44] Ahmed BY, Husnain O, Stafford R, Howard M, Gujar AS, Moradiya V, Patel KK, Sihotra S (2013). Hyperphosphorylation of CREB in human dopaminergic neurons: a kinetic study of cellular distribution of total CREB and phospho-CREB following oxidative stress. Neuroreport.

[45] Mimura K, Ando T, Poschke I, Mougiakakos D, Johansson CC, Ichikawa J, Okita R, Nishimura MI, Handke D, Krug N, Choudhury A, Seliger B, Kiessling R (2011). T cell recognition of HLA-A2 restricted tumor antigens is impaired by the oncogene HER2. Int J Cancer.

[46] Straino S, Germani A, Di Carlo A, Porcelli D, De Mori R, Mangoni A, Napolitano M, Martelli F, Biglioli P, Capogrossi MC (2004). Enhanced arteriogenesis and wound repair in dystrophin-deficient mdx mice. Circulation.

[47] Bukur J, Herrmann F, Handke D, Recktenwald C, Seliger B (2010). Identification of E2F1 as an important transcription factor for the regulation of tapasin expression. J Biol Chem.

[48] Tal MC, Sasai M, Lee HK, Yordy B, Shadel GS, Iwasaki A (2009). Absence of autophagy results in reactive oxygen species-dependent amplification of RLR signaling. Proc Natl Acad Sci U S A.

[49] Robles-Martinez L, Guerra-Sanchez MG, Flores-Herrera O, Hernandez-Lauzardo AN, Velazquez-Del Valle MG, Pardo JP (2013). The mitochondrial respiratory chain of Rhizopus stolonifer (Ehrenb.:Fr.) Vuill. Arch Microbiol.

[50] Li Q, Birkbak NJ, Gyorffy B, Szallasi Z, Eklund AC (2011). Jetset: selecting the optimal microarray probe set to represent a gene. BMC Bioinformatics.

[51] Zhang J, Ye J, Ma D, Liu N, Wu H, Yu S, Sun X, Tse W, Ji C (2013). Cross-talk between leukemic and endothelial cells promotes angiogenesis by VEGF activation of the Notch/Dll4 pathway. Carcinogenesis.

