# Supplementary material for: Hypoxia-mediated alterations and their role in the HER-2/neuregulated CREB status and localization
Source: Oncotarget. 2016 Jul 7;7(32):52061–84. doi: 10.18632/oncotarget.10474 (PMC5239535; doi:10.18632/oncotarget.10474)
Supplement: Supplementary file 1 [file oncotarget-07-52061-s001.pdf]

Hypoxia-mediated alterations and their role in the HER-2/neu-regulated CREB status and localization

SUPPLEMENTARY FIGURES AND TABLE

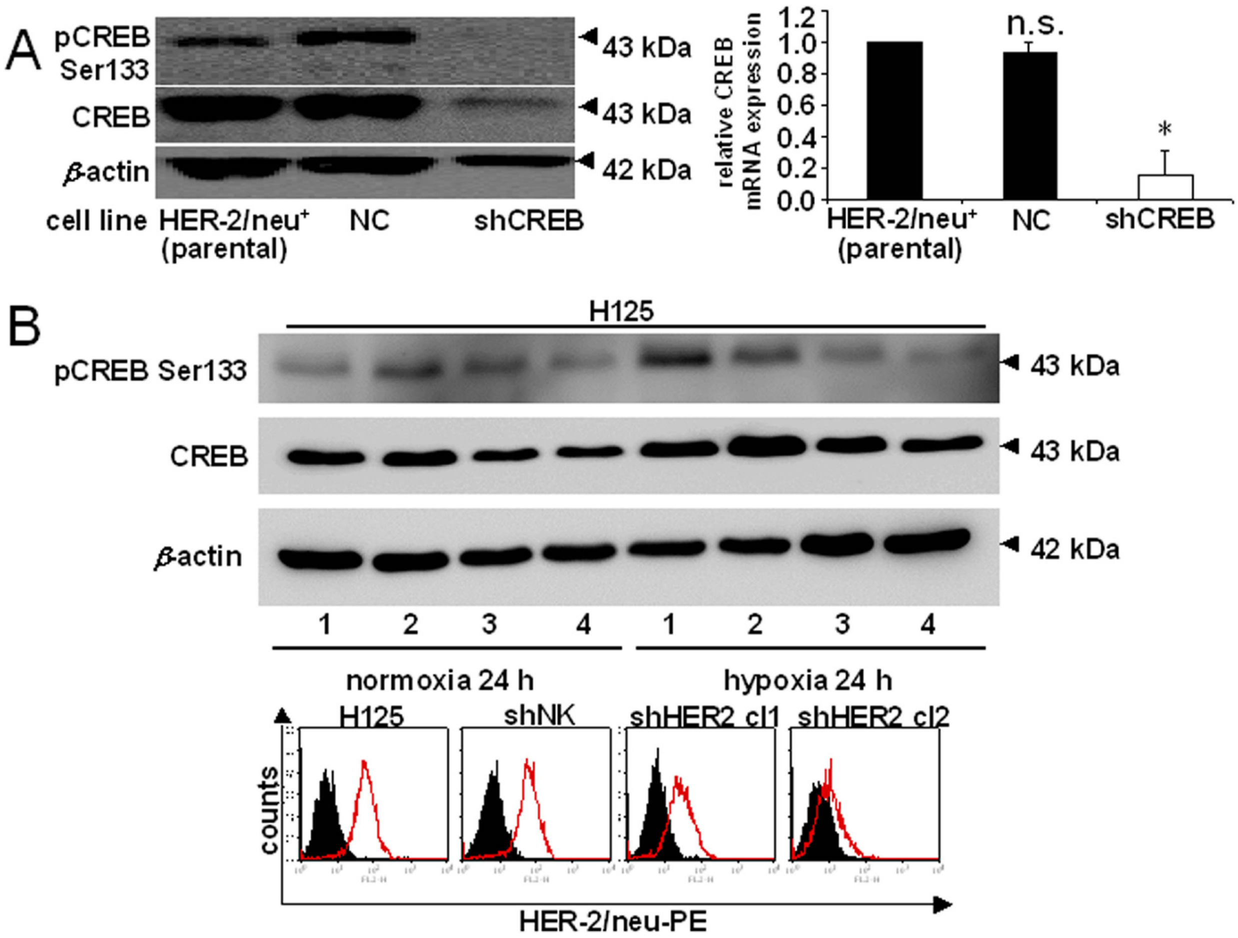

Supplementary Figure S1: Effect of shCREB and hypoxia on murine fibroblasts and lung cancer cells. A. 50 µg protein lysate of parental HER-2/neu⁺ cells and stable transfectants carrying a non-sense (NC) shRNA or a CREB-specific shRNA (CREB-deficient HER-2/neu⁺ cells) was subjected to Western blot analysis as described in Materials and Methods using an anti-CREB and anti-pCREB^{Ser133} mAb, respectively, while staining with an anti-β-actin mAb served as a loading control. The blot represents one of three biological replicates (left). mRNA was isolated from parental HER-2/neu⁺ cells and transfectants and subjected to quantitative qPCR analysis as described in Materials and Methods. The expression was normalized to β-actin expression as a house keeping gene. The bar charts represent the mean and SEM from three biological replicates (right). **B.** The CREB protein expression and phosphorylation in H125 cells and derived cell lines under 24 h normoxia or hypoxia was analysed by Western Blot. 50 µg total protein lysate was loaded upon a SDS gel and were separated. Beta actin was used as a loading control. The blots represent one of two independent experiments. The lanes are the following cell lines: 1 = H125 cell line, 2 = H125 shNK (non-sense control), 3 = H125 shHER2 cl1 (HER-2/neu knock down clone 1), 4 = H125 shHER2 cl2 (HER-2/neu knock down clone 2). The flow cytometric histograms show the surface expression of HER-2/neu (black area) compared to a IgG control (red lined area) from one of two independent experiments. shNK represents cells transfected with a non-sense control plasmid, while shHER2 cl1 and shHER2 cl2 are two independent clones transfected with a shRNA plasmid targeting HER-2/neu.

(*Continued*)

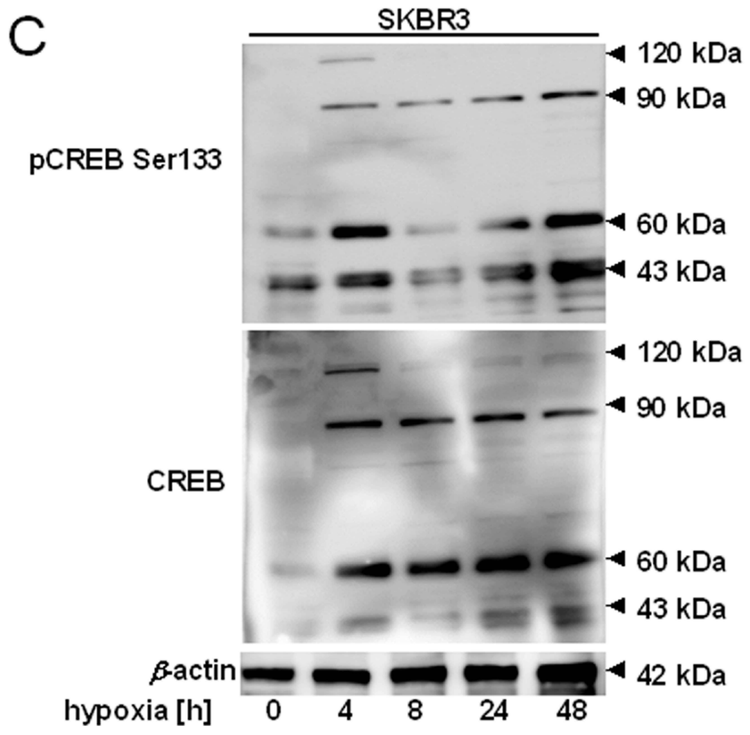

Supplementary Figure S1 (*Continued*): C. SKBR3 cells were incubated under normoxia or hypoxia for the indicated time. The phosphorylation of CREB, ERK and total CREB or ERK were measured with specific antibodies. Beta-Actin served as the loading control.

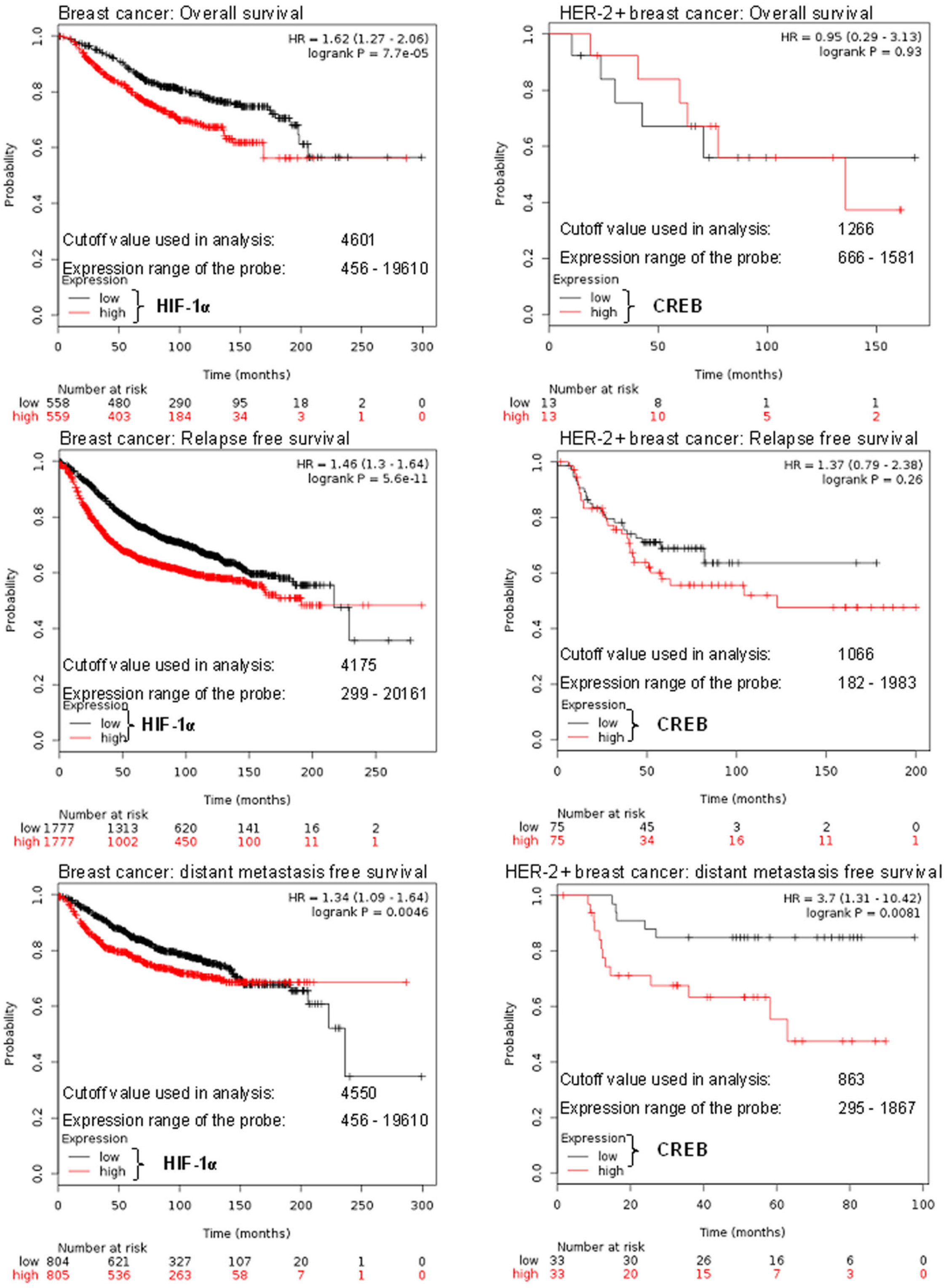

Supplementary Figure S2: Kaplan-Meier plots of breast cancer patients. The effect of CREB and HIF-1α overexpression in HER-2+ breast cancer patients on the overall survival, relapse free survival, and distant metastasis free survival was analysed by the Kmplot.com site using the affy ID “225572_at” for CREB1 and “200989_at” for HIF-1α.

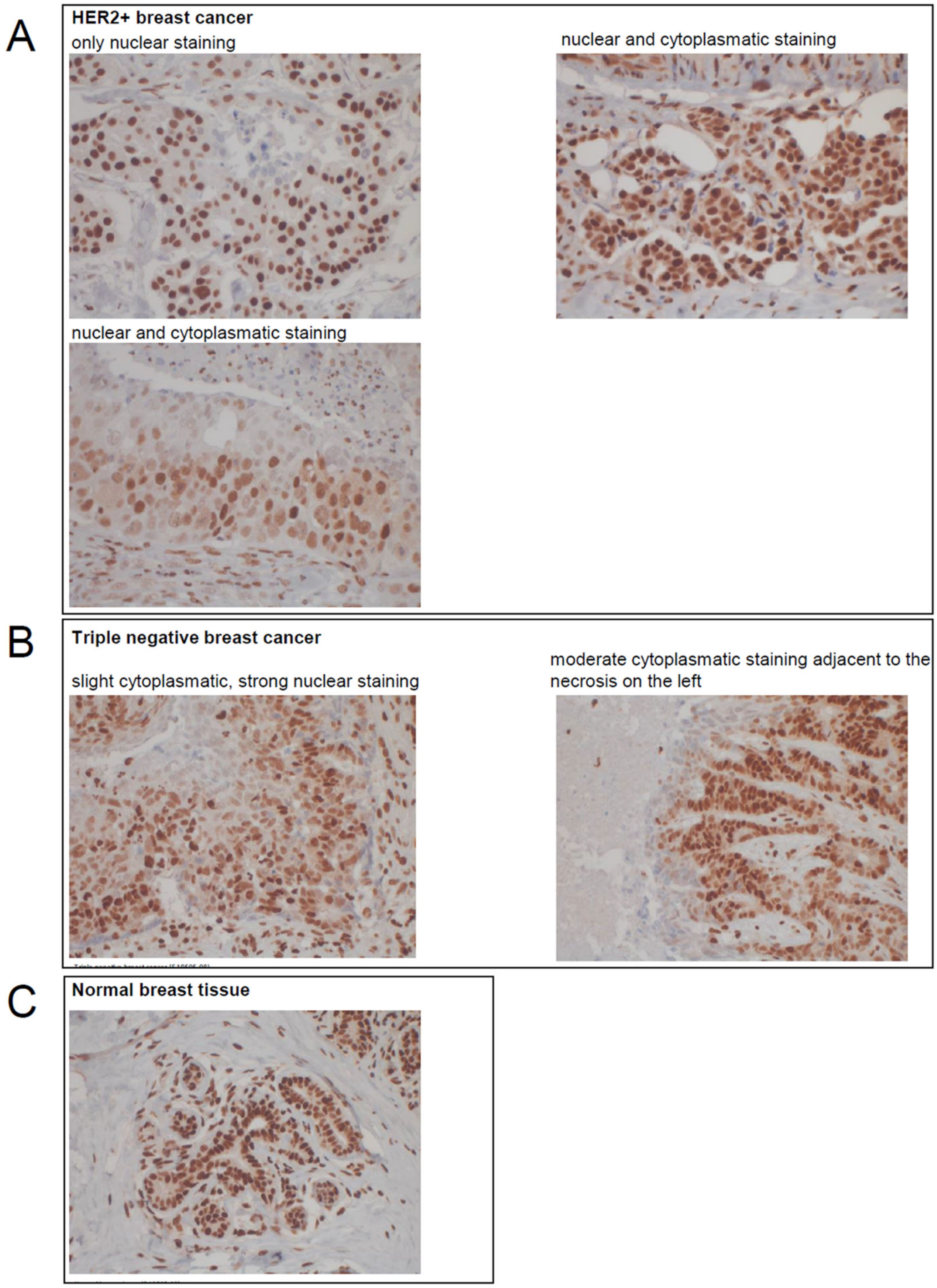

Supplementary Figure S3: *in situ* localization of CREB in hypoxic tissue. Breast cancer tumor lesions of HER-2^{+} and triple negative breast cancer were stained with p-CREB antibody (Ser133, 87G3, dilution: 1:200) and were compared with normal breast tissue. The pathological findings are listed above the picture.

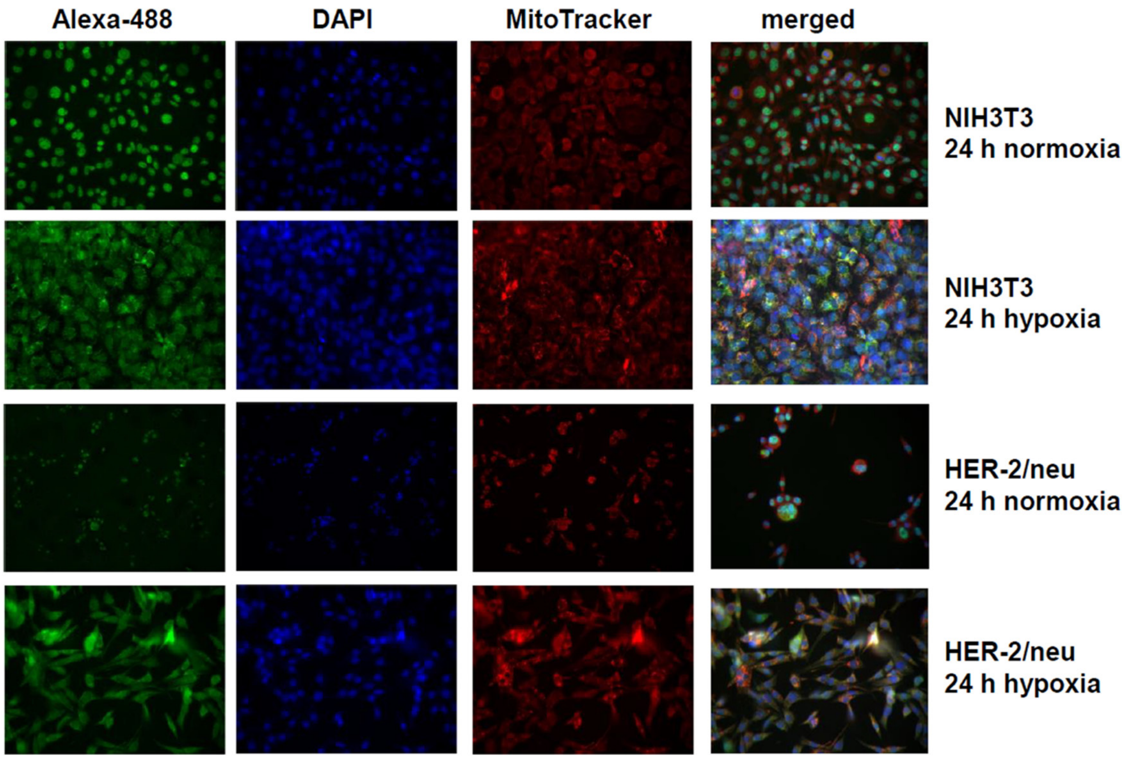

Supplementary Figure S4: Subcellular localization of CREB under normoxia and hypoxia (unmerged pictures from Figure 6D). Cells were incubated for 24 h under normoxia or under hypoxia and the localization of CREB was analysed by immunocytochemical staining. Alexa 488 represents the CREB staining, while mitochondria were stained with MitoTracker Red and DAPI was used for nuclear staining. The adhesion potential of HER-2/neu cells under normoxic conditions is lower than under hypoxic conditions, resulting in a decreased cell number.

Supplementary Table S1: Survival of HER-2/neu positive breast cancer patients with CREB overexpression

Affymetrix ID (CREB1)	OS St Gallen molecular subtype HER2^{+} (**)	DMFS St Gallen molecular subtype HER2^{+} (**)	RFS St Gallen molecular subtype HER2^{+} (**)	OS HER2 positive (***)	DMFS HER2 positive (***)	RFS HER2 positive (***)
237289_at	0.16	0.38	0.41	0.31	0.53	0.85
204314_s_at	0.6	0.082	0.99	0.97	0.037	0.036
225572_at (*)	0.33	0.29	0.61	0.93	0.0081	0.26
214513_s_at	0.062	0.41	0.039	0.24	0.23	0.66
204312_x_at	0.38	0.3	0.15	0.7	0.71	0.83
204313_s_at	0.88	0.15	0.15	0.31	0.023	0.27
225565_at	0.64	0.051	0.000003	0.76	0.00019	0.000017

* - JetSet best score (Supplementary Figure 3)
** - measured by gene chip
*** - measured by IHC / FISH
Overall survival (OS), distant metastasis free survival (DMSF) and relapse free survival (RFS) in breast cancer patients with an increased expression of HER-2/neu (Subgroup “HER2 status: HER2 positive”) or with the HER2+ St Gallen molecular subtype (Subgroup “intrinsic subtype: HER2+”) was analysed under influence of an overexpression of CREB1. A logrank $P > 0.05$ is not significant (grey). Red indicates a worser prognosis with CREB1 overexpression, while green an increast survival with CREB 1 overexpression. The CREB1 Affy ID with the best JetSet Score (rating the probe sets for specificity, coverage and degradation resistance), that was used for the Kaplan Meier curves in Supplementary Figure 3, is highlighted in blue.
